# Bioactivities of Quinic Acids from *Vitex rotundifolia* Obtained by Supercritical Fluid Extraction

**DOI:** 10.3390/antiox13101235

**Published:** 2024-10-14

**Authors:** Duc Dat Le, Young Su Jang, Vinhquang Truong, Soojung Yu, Thientam Dinh, Mina Lee

**Affiliations:** 1 College of Pharmacy and Research Institute of Life and Pharmaceutical Sciences, Sunchon National University, 255 Jungangno, Suncheon 57922, Jeonnam, Republic of Korea; ddle@scnu.ac.kr (D.D.L.); quangvtruong00@gmail.com (V.T.); thientamm.2001@gmail.com (T.D.); 2 Nano Bio Research Center, Jeonnam Bio Foundation, Jangseong 57248, Jeonnam, Republic of Korea; ysjang@jbf.kr; 3 Department of Natural Cosmetics Science, Graduate School, Sunchon National University, 255 Jungangno, Suncheon 57922, Jeonnam, Republic of Korea; ysj1997s@naver.com

**Keywords:** *Vitex rotundifolia*, anti-inflammatory, antioxidant, MAKP, iNOS, COX-2

## Abstract

Acyl-quinic acids (AQAs), present in various plants with many health benefits, are regarded as therapeutic agents in the prevention and treatment of chronic and cardiovascular diseases. The molecular network-guided identification of ten AQA compounds, two new (**5** and **7**) and eight known compounds, were isolated from *V. rotundifolia* L. f. by using a newly applied extraction method. Their structures were determined through spectroscopic means, reaction mixtures, and modified Mosher and PGME techniques. These compounds were assessed for their anti-inflammatory and antioxidant capabilities. Notably, compounds **1**, **3**, **4**, **6**, **8**, and **9** exhibited notable DPPH radical scavenging activity. In LPS-induced HT-29 cells, compounds **2**–**7** significantly inhibited IL-8 production. Furthermore, compounds **3**–**5** and **7** markedly suppressed NO production, while compounds **1**–**10** effectively inhibited IL-6 production in LPS-induced RAW264.7 cells. Western blot analyses revealed that compounds **3**–**5**, and **7** reduced iNOS and COX-2 expression, and compounds **2**–**5**, **7**, and **8** also diminished the expression levels of p38 MAPK phosphorylation. Docking studies demonstrated the active compounds’ binding affinity with the IL-8, iNOS, COX-2, and p38 MAPK proteins through interactions with essential amino acids within the binding pockets of complexes. The findings suggest that compounds **1**, **3**, **4**, **6**, **8**, and **9**, and compounds **3**–**5**, and **7**, hold promise as potential therapeutic agents for treating antioxidative and inflammatory diseases, respectively.

## 1. Introduction

The inflammatory process is a protective response by the body to stimuli, aimed at repairing damaged tissue, restoring homeostasis, and eradicating pathogens [1]. During an inflammatory state, a plethora of pro-inflammatory mediators and cytokines are released, synthesized either by cells at the site of inflammation or by the liver for circulation in the blood plasma. These chemical substances can be activated upon contact with receptors at the sites of inflammation [2], initiating numerous intracellular signaling pathways. Consequently, the overproduction of these mediators and cytokines leads to toxic effects on host cells and tissues during the signaling process. Therefore, identifying anti-inflammatory inhibitors that can protect cells and tissues from damage or differentiation by controlling the rate of production of inflammatory mediators in balance with the immune system is crucial.

Natural materials are processed to extract bioactive compounds from food plants by traditional methods including Soxhlet distillation, hot extraction, maceration, and sonification. However, these methods have some disadvantages, such as low efficiency and environmental pollution due to usage of large amounts of toxic solvents or long-term extraction [3]. Therefore, it is necessary to develop new extraction methods for more advanced techniques and lower toxicity. Among them, supercritical fluid extraction (SFE) can adapt many advantages to requirements and be widely employed in the food-based industries [4]. SFE is known as a green technology with several advantages providing higher efficiency and shortened time of extraction. This method mainly uses carbon dioxide (CO_2_) as a single solvent or co-solvent by mixing with other solvents. The SFE method may be controlled by the optimization of process parameters, including temperature, pressure, and the ratio of co-solvent corresponding to phytochemicals on targeting extracts [5].

*Vitex rotundifolia* L. f. is known as the coastal medicinal plant and extensively found in tropical and temperate regions of Australia, Southeast Asian countries, and Pacific Islands [6]. It has been traditionally used alone or in combination with other plants and has a long history of use for treating a variety of diseases, such as hair loss, headaches, fever, wound recovery, traumatic injuries, eye pain, swelling, bruises, in the pharmacopoeias and traditional folk medicines of many countries [7,8,9]. Therefore, this folk medicine is extensively applied in clinical trials targeting various diseases, such as the Qiwei Ketengzi pill, the Huangu pill, and Dieda Wanhua oiling agent [10]. This plant has also revealed a broad range of bioactivities including antioxidative, anti-inflammatory, analgesis, and anticancer capacities [11]. The potential medicinal benefits and diverse pharmacological activities may contain a pool of metabolites with potential complementary therapeutic actions.

*V. rotundifolia* has the potential for many valuable activities in traditional medicine. Thus, finding the precise active components of this folk medicine is crucial to highlight its anti-inflammatory benefits. In the present study, we aim to investigate the anti-inflammatory property of this folk medicinal plant by employing an advanced technique for extraction for the first time. The chemical structures of isolates were established by the proposed effective methods for determining the structural configurations, employing 1D selective NOE, 1D/2D NMR, Mosher, and PGME techniques. The isolates showed good anti-inflammatory capacity by inhibiting the product expression of cytokines, mediators, and enzymatic proteins as well as reducing phosphorylation of p38 MAPK signaling.

## 2. Materials and Methods

### 2.1. Plant Materials

At Shinan (Jeonnam, Republic of Korea), the twigs of this plant were collected and identified by Professor Mina Lee (College of Pharmacy, Sunchon National University). After that, a voucher specimen was made and stored at the Pharmacognosy Laboratory (College of Pharmacy, Sunchon National University) in Korea.

### 2.2. Fractionation and Separation of Marker Compounds ***1***–***7***

The sample was pre-treated and extracted using a supercritical fluid extraction system. Before injecting the sample into the equipment, the temperature was set at 50 °C. Once the equipment’s temperature stabilized, the sample (8000 g) was injected into the system using a high-pressure pump. Subsequently, a fixed amount of CO_2_ was injected until it reached 400 bar. During the gas injection, the valve was adjusted to ensure that the pressure inside the equipment changed linearly over time and was maintained at a constant level. Ethanol was added as a co-solvent with a rate of 5 mL/120 min. Then, solution extract was concentrated under vacuum to obtain a residue of 400 g. This residue was subsequently suspended in distilled water and then partitioned using increasing solvent polarity from *n*-hexane to *n*-BuOH to obtain *n*-hexane (H), methylene chloride (MC), EtOAc (E), *n*-BuOH (B), and water fractions (W), respectively. Then, the EtOAc fraction was further separated by using multiple chromatographic techniques (Supplementary Materials) to obtain ten isolated compounds (**1**–**10**).

### 2.3. LC-MS/MS Conditions and Molecular Network Experiments

The total extract and the H, MC, E, Bu, and W fractions were dissolved in MeOH and filtered through polytetrafluoroethylene membrane filters before injection for LC-MS/MS analysis. The LC-MS/MS system included a Vanquish UHPLC system decoupled with an Orbitrap Exploris 120 mass spectrometer (Thermo Fisher Scientific, Sunnyvale, CA, USA). The LC was performed using a Waters Acquity UPLC HSS T_3_ column (4.6 × 100 mm, 1.8 μm, Waters, Milford, MA, USA) at 40 °C with a flow rate of 0.4 mL/min, and an injection volume of 4 μL. Mobile phase elution consisted of a gradient solvent system with phase A (pure water containing 0.1% formic acid) and phase B (ACN, 0.1% formic acid) as follows: 5–15% (B) for 0–4 min, 15–35% (B) for 4–12 min, 35–45% (B) for 12–17 min, 45–100% (B) for 17–23 min and held for three minutes, and 100–5% (B) for 1 min before re-equilibrium with 5% (B).

### 2.4. Feature-Based Molecular Networking (MN)

**Figure 1:**
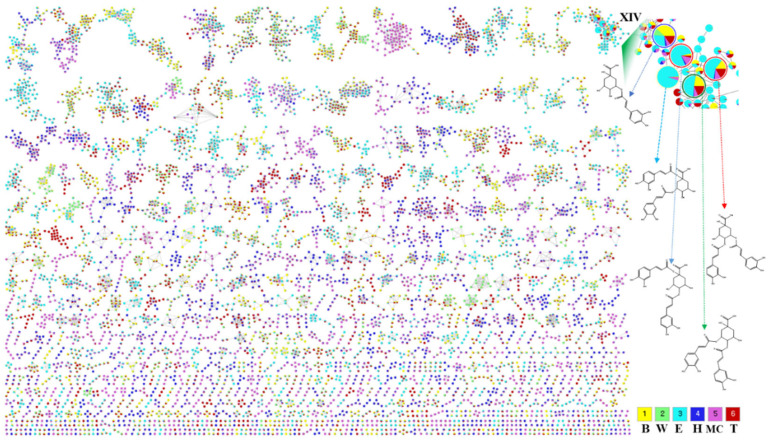
Feature-based molecular network of total extract (T, Red) and *n*-BuOH (B, Yellow), water (W, Glass), EtOAc (E, Tiffany blue), *n*-hexane (H, Dark blue), and methylene chloride (MC, Purple) fractions of *Vitex rotundifolia*.

The molecular network (Figure 1) was performed using the GNPS web platform (https://gnps.ucsd.edu accessed on 18 July 2024), files mgf and quant. CSV formats were obtained by using Mzmine 3.9.0 and were then uploaded to the GNPS web tool to generate a feature-based molecular networking file.graphml. MS spectral data were searched against the GNPS spectral library. The MN was visualized with Cytoscape 3.9.1 (https://www.cytoscape.org/ accessed on 18 July 2024). The workflow can be found in the following GNPS (https://gnps.ucsd.edu accessed on 18 July 2024) repository with a task ID of c8198cc917fa4f03aeb0c4534a7e6746.

### 2.5. Modified Mosher Method

The modified Mosher method [12] was applied to determine the absolute configuration at C-3 (**7**) and C-4 (**5**). Briefly, 0.8 mg for each was added to 300 µL of anhydrous pyridine. Then, (*S*)-(+)-α-methoxy-α-(trifluoromethyl)phenylacetylchloride (MTPACl) (15 µL) or (*R*)-MTPACl) (15 µL) was implemented. After stirring for 24 h, these reaction mixtures were collected at room temperature. Each mixture (**5** with **5a** and **5b**; **7** with **7a** and **7b**) was dried to dryness before injecting into the LC-MS system to confirm the reaction products. After the reaction was completed, they were purified by preparative HPLC to afford **5a** and **5b**, and **7a** and **7b** (Scheme S1, Supplementary Materials). The ∆*δ*_S-R_ values around the stereogenic centers of the MTPA diesters were determined by analysis of NMR spectra (Figure 1).

### 2.6. Preparation of (S)- and (R)-Phenylglycine Methyl Ester (PGME) Amides of ***5***

A PGME method was applied to determine C-1 configuration by following a previous report [13] without modifications (Scheme S2 and Figure S1, Supplementary Materials).

### 2.7. Antioxidative Assay

All isolated compounds (**1**–**10**) and positive control (ascorbic acid, AA), were prepared at the concentrations of 10 and 100 µM. An experimental assay to evaluate the DPPH radical scavenging activity was applied as per our previous assay [14]. Briefly, the DPPH assay was used to assess antioxidant activity. DPPH (0.2 mM in ethanol) solution was prepared. Samples were diluted to 10 µM in ethanol. Ascorbic acid (10 and 100 µM) served as a positive control. Scavenging activity (%EC) was calculated as (Acontrol − Asample)/Acontrol × 100. Absorbance was measured at 517 nm by using a microplate reader (Bio Tek Instruments, Winooski, VT, USA).

### 2.8. Cell Culture and Cell Viability of Human Colon Epithelial (HT-29) and Mouse Macrophage (RAW264.7) Cells

Compounds **1**–**10** were prepared at concentrations of 10 and 100 µM. RAW264.7 and HT-29 cells were cultured by using a similar method. They were maintained by adding 10% heat-inactivated FBS, penicillin (100 IU/mL), and streptomycin sulfate (100 µg/mL), to Dulbecco’s modified Eagle’s medium. Then, the mixture solution was incubated under normoxic conditions in a humidified environment at 37 °C with 5% CO_2_. RAW264.7 cells were incubated for 24 h and then they were implemented with compounds. After 1 h, cells were stimulated with 1 µg/mL LPS before incubating for 16 h in the 96-well plates. After 2 h, they were stimulated with LPS (100 ng/mL) for 12 h.

Similarly, HT-29 cells were incubated for 24 h, stimulated with LPS for 12 h, and they then were treated with compounds for 2 h in the 96-well plates. The cell survival rate was evaluated by MTT assay, operating the absorbance at 570 nm using a microplate reader (Bio Tek Instruments, Winooski, VT, USA).

### 2.9. Determination NO Production

Compounds **1**–**10** and the positive control (L-NG-nitroarginine methyl ester, _L_-NAME) were prepared at concentrations of 10 and 100 µM. The nitrite secreted from cell supernatant in LPS-stimulated RAW264.7 cells was conducted using our previous method [15] without modification. Experiments were designed to measure NO production at 550 nm by using a microplate reader.

### 2.10. ELISA Assay

To assess the production of IL-6 and IL-8, we used RAW264.7 cells (5 × 10^4^ cells/well) and HT-29 cells (3 × 10^5^ cells/well), respectively. The cells were seeded in 96-well plates overnight for cell adherence and pre-incubated in the presence or absent of compounds for 2 h. After stimulating with LPS (100 ng/mL) for the next 24 h, supernatant was collected. IL-6 and IL-8 production was detected by ELISA kit following the manufacturer’s instructions.

### 2.11. Western Blot Assay

After pre-treating with compounds, RAW264.7 cell lines were lysed with PRO-PREPTM (Intron Biotechnology, Seoul, Republic of Korea) supplemented. Protein concentrations were determined with a Pierce™ Bradford Protein Assay Kit (Thermo Fisher Scientific) according to the manufacturer’s instructions. Whole proteins (20 μg) were separated by SDS-PAGE using a 10% acrylamide gel and then transferred to a PVDF membrane. Antibodies including p-p38 (#9211) and p38 (#9212) MAPK were procured from Cell Signaling Technology (Danvers, MA, USA). Actin (AB_2289199), iNOS (AB_397808), and COX-2 (AB_397603) were purchased from BD Bioscience (San Jose, CA, USA). Blots were incubated with these antibodies diluted at 1:1000 in 2.5% skimmed milk. After incubation overnight at 4 °C for 18 h, blots were washed three times with Tween20/Tris-buffered saline (T/TBS) and incubated with an HRP-conjugated secondary antibody diluted 1:2000 in 5% skimmed milk for 2 h at room temperature and then washed three times again using T/TBS. These membranes were developed using a Super Signal™ West Femto Maximum Sensitivity Substrate (Thermo Fisher Scientific, Waltham, MA, USA).

### 2.12. In Silico Studies

The crystal structures of target proteins such as IL-8 (PDB ID: 5D14), iNOS (PDB ID: 3E7G), COX-2 (PDB ID: 5IKQ), and p38 MAPK (PDB ID: 1A9U) were retrieved from the RCSB protein data bank (https://www.rcsb.org; accessed on 18 July 2024). Each protein was added to the MGL tools 1.5.6. Then, it was processed by removing water and heteroatoms and adding polar hydrogen atoms and Kollman charges. After the final step of receptor preparation, the protein was saved in pdbqt format. The structures of compounds were downloaded from PubChem (https://pubchem.ncbi.nlm.nih.gov, 18 July 2024) in sdf formats, whereas the missing structures were drawn out by using the Avogadro (version 1.2) package. Subsequently, the Open Babel program was utilized to convert them into their respective pdbqt formats. After that, each structure was added to MGL tools 1.5.6 as ligands before adding Gasteiger charges. The grid box parameters were established by using a Pymol program. The Lamarckian genetic algorithm in AutoDock 4.2.6 was applied to approach the best conformation of the ligands. The resulting complexes were established and visualized by using a Discovery Studio Visualizer 2021 and Pymol programs.

### 2.13. Statistical Analysis

Data were represented as the means ± standard deviations (S.D.) of three replicates. The nonparametric two-way ANOVA followed by Dunnett’s multiple comparison test using the Graphprism version 8.0.1 software (Graphpad Software, La Jolla, CA, USA) was used for statistical analyses.

## 3. Results

### 3.1. Molecular Network-Guided Isolation of AQA Compounds

**Figure 2:**
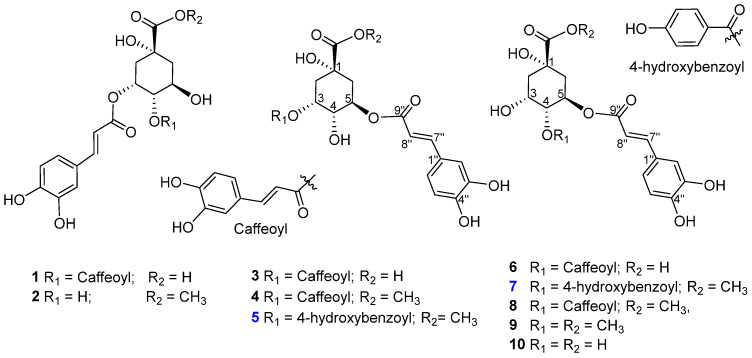
Chemical structures of two new (**5** and **7**, blue highlight) and eight known (**1**–**4**, **6**, **8**–**10**) isolated compounds from *V. rotundifolia*.

AQAs were naturally synthesized by esterification between quinic acid and hydroxycinnamic acids, such as p-coumaric, caffeic, sinapic, and ferulic acids through different biosynthesis pathways. Depending on the number of substituents attached, these compounds can be categorized into mono-, di-, tri-, and tetra-substituents with quinic acids. They are derived from various parts of plants and foods, including fruits, vegetables, coffee, and spices [16]. Among the chemical constituents isolated from *V. rotundifolia*, quinic acid is one of the major groups found in this plant. To separate target AQA compounds, the total extract and fractions derived from *V. rotundifolia* were conducted by using an LC-MS system. Then, a feature-based molecular networking approach was applied for mapping and identifying the chemical structures in non-targeted mass spectrometry data in cooperation with the GNPS platform to generate chromatographic feature detection and alignment. As shown in the below Figure 2, cluster XIV showed nodes having AQA structures which were identified by using in silico GNPS tools in combination with online GNPS data. These AQAs were relatively determined with some plural amounts from EtOAc and Bu fractions (Figure 1). Thus, these fractions were selected as materials for isolating AQA compounds.

### 3.2. Structural Elucidation

Two new compounds (**5** and **7**) and eight known ones were isolated from *V. rotundifolia* by using multiple chromatographic techniques. Their structures were identified as 3,4-di-*O*-caffeoylquinic acid (**1**) [17], 3,4-di-*O*-caffeoylquinic acid methyl ester (**2**) [18], 3,5-di-*O*-caffeoylquinic acid (**3**), 3,5-di-*O*-caffeoylquinic acid methyl ester (**4**) [19], 4,5-di-*O*-caffeoylquinic acid (**6**) [20], 4,5-di-*O*-caffeoylquinic acid methyl ester (**8**) [21], 5-*O*-caffeoylquinic acid methyl ester (**9**), and chlorogenic acid (**10**) [19] (Figure 2). This determination occurred through analysis of their spectroscopic data and comparison with those reported in the literature.

**Figure 3:**
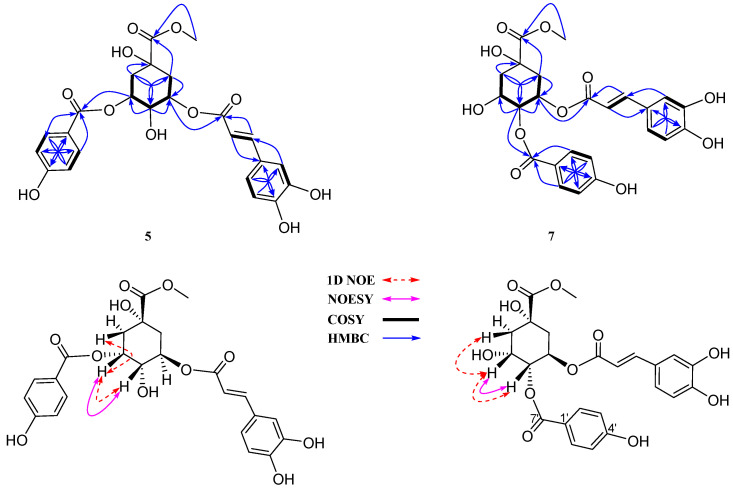
Key HMBC (blue arrow), COSY (black line), selective 1D NOE (red-dash arrow), and NOESY (magnetic arrow) correlations of compounds **5** and **7**.

Compound **5**, obtained as a light yellowish amorphous powder, exhibited a deprotonated ion [M–H]^−^ peak at *m*/*z* 487.1245 (calculated for C_24_H_23_O_11_, 487.1246) in the HR-ESIMS spectrum. Also, high mass data revealed fragmentation of a benzoyl moiety at *m*/*z* 137.0246 and a caffeoyl fragment at *m*/*z* 161.0246 (Figure S2, Supplementary Materials). The ^1^H NMR spectrum presented key signals: an A_2_B_2_ spin system at *δ*_H_ 7.96 (2H, d, *J* = 8.7 Hz, H-2′/6′) and 6.82 (2H, d, *J* = 8.7 Hz, H-3′/5′), a *trans*-olefinic coupling at *δ*_H_ 7.54 (1H, d, *J* = 16.0 Hz, H-7″) and 6.22 (1H, d, *J* = 16.0 Hz, H-8″), an ABX spin system at *δ*_H_ 7.04 (1H, d, *J* = 2.3 Hz, H-2″), 6.96 (1H, dd, *J* = 2.3, 8.2 Hz, H-6″), and 6.77 (1H, d, *J* = 8.2 Hz, H-5″), three oxygenated methines at *δ*_H_ 5.48 (H-3), 5.35 (H-5), and 4.00 (H-4), a methoxy proton at *δ*_H_ 3.68, and four protons belonging to two methylene groups at *δ*_H_ 2.35 (H-2b), 2.20 (H-6), and 2.18 (H-2a). The ^13^C NMR spectrum revealed signals for three carboxylic groups at *δ*_C_ 175.8 (C-7), 168.1 (C-9″), and 167.7 (C-9′) (Figures S3 and S4, Supplementary Materials); eight unsaturated carbons; a quinic moiety; six remaining carbons; and a methoxy group at *δ*_C_ 53.0 (Table 1). The HMBC cross-peaks from H-2′/6′ (*δ*_H_ 7.96) and H-3 (*δ*_H_ 5.48) to C-7′ (*δ*_C_ 167.7) (Figure 3) illustrated the combination of the *p*-hydroxybenzoyl moiety with the quinic skeleton at the C-3 position. Additionally, COSY correlations between H_2_-2/H-3/H-4/H-5/H_2_-6 and the HMQC assignments of their corresponding carbons constructed the quinic moiety (Figure 3 and Figures S5–S7, Supplementary Materials). The configuration of structure **5** was deduced by interpreting NMR data and identifying correlations in 1D NOE and 2D NOESY spectra, alongside the application of the PGME and modified Mosher methods. Crucially, a series of Δ*δ*_SR_ values (Figures S1, S10 and S11, Supplementary Materials), representing the differential chemical shifts for analogous proton pairs between *S*- and *R*-MTPA diastereomeric esters, validated the C-4*S* configuration of the quinic moiety. A pronounced NOESY correlation between H-3 and H-4 indicated their *β*-orientation (Figure S8, Supplementary Materials). Selective 1D NOE irradiation of H-4 significantly enhanced the signal for H-3. Irradiating H-3 heightened the signals at H_2_-2 and H-4, which were enhanced without the effect of H-5 (Figure S9, Supplementary Materials). This observation approved for the H-3β, H-4β, H-5α configurations and assignment of H_2_-2/H_2_-6, absolutely. Furthermore, acid hydrolysis of compound **5** led to the identification of a quinic acid moiety, followed by a PGME method to determine the C-1*R* configuration. Δ*δ* values of the (*S*)- and (*R*)-PGME amides **5c** and **5d** (Scheme S2 and Figures S12 and S13, Supplementary Materials), confirmed the C-1 configuration. Finally, the structure of compound **5** was conclusively determined as a new compound with the trivial name rotundi A.

Compound **7** is presented as a light yellowish amorphous powder. Its HR-ESIMS spectrum revealed a deprotonated ion [M–H]^−^ peak at *m*/*z* 487.1244 (calculated for C_24_H_23_O_11_, 487.1240) (Figure S14, Supplementary Materials). The 1H NMR spectrum of **7** exhibited characteristic signals of an aromatic A_2_B_2_ spin system at *δ*_H_ 7.92 (2H, d, *J* = 8.8 Hz, H-2′/6′) and 6.81 (2H, d, *J* = 8.8 Hz, H-3′/5′); an ABX spin system at *δ*_H_ 6.99 (1H, d, *J* = 1.0 Hz, H-2″), 6.91 (1H, dd, *J* = 1.0, 8.2 Hz, H-6″), and 6.76 (1H, d, *J* = 8.2 Hz, H-5″); trans-oriented olefinic protons at *δ*_H_ 7.48 (1H, d, *J* = 16.0 Hz, H-7″) and 6.14 (1H, d, *J* = 16.0 Hz, H-8″); three methines at *δ*_H_ 5.60 (1H, m, H-5), 5.18 (1H, dd, *J* = 3.1, 8.0 Hz, H-4), and 4.41 (1H, m, H-3) alongside two methylene groups at *δ*_H_ 2.37 (1H, dd, *J* = 3.7, 14.1 Hz, H-2a), 2.09 (1H, dd, *J* = 6.9, 14.1 Hz, H-2b), 2.30 (1H, dd, *J* = 4.9, 8.7 Hz, H-6a), and 2.23 (1H, dd, *J* = 4.6, 13.7 Hz, H-6b); and a methoxy group at *δ*_H_ 3.71 (3H, s) (Figure S15, Supplementary Materials).

**Figure 4:**
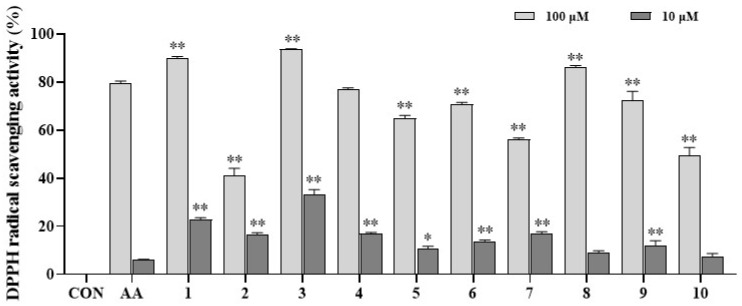
Antioxidant effect of compounds **1**–**10** and ascorbic acid (AA). DPPH assay was performed in triplicates. Differences were significant at * *p* < 0.01 and ** *p* < 0.001, compared to positive control (ascorbic acid, AA) for each concentration of 10 µM or 100 µM.

The ^13^C NMR spectrum of **7** detected 24 carbon signals including three carboxy resonances at *δ*_C_ 175.2 (C-7), 167.5 (C-7′), and 167.9 (C-9″); aromatic carbons (Figure S16, Supplementary Materials); two olefinic carbons; a methoxy group; and the remaining six in a quinic moiety (Table 1). The ^1^H−^1^H COSY correlations of H_2_-2 (*δ*_H_ 2.09/2.37)/H-3 (*δ*_H_ 4.41)/H-4 (*δ*_H_ 5.18)/H-5 (*δ*_H_ 5.60)/H_2_-6 (*δ*_H_ 2.23/2.30) were observed (Figure 4).

Upon selective 1D NOE spectroscopy, irradiation of H-3 enhanced signals for H_2_-2 and H-4, while light signal enhancement of H-4 and H_2_-6 was observed upon irradiation of H-5. This elucidated the accurate signal assignments for these neighboring protons (Figure 4). The above information together with ^1^H–^13^C HMQC assignment established the quinic skeleton (Figure 3). The low-field resonances of two oxymethine protons at H-4 (*δ*_H_ 5.18) and H-5 (*δ*_H_ 5.60) suggested the attachment of the benzoyl and caffeoyl ester groups at these positions, respectively. Indeed, the HMBC correlation of H-2′/6′ (*δ*_H_ 7.92) and H-4 (*δ*_H_ 5.18) to C-7′ (*δ*_C_ 167.5), indicated the combination of the *p*-hydroxybenzoyl moiety with the quinic skeleton through the C-4 position. HMBC correlations of H-7″ (*δ*_H_ 7.48) to C-2″ (*δ*_C_ 115.0)/C-6″ (*δ*_C_ 121.7)/C-9″ (*δ*_C_ 168.1), and a long-range HMBC correlation of H-5 (*δ*_H_ 5.60) to C-9″ (*δ*_C_ 168.1) (Figure 4), confirmed the attachment of the caffeoyl moiety through the C-5 position (Figures S17–S19, Supplementary Materials). Moreover, the HMBC cross-peak of a methoxy group at *δ*_H_ 3.71 to C-7 (*δ*_C_ 175.2) established the 7-OCH_3_ arrangement (Figure 3).

The relative configuration of **7** was deduced through 1D NOE and 2D NOESY (Figures S20 and S21, Supplementary Materials) correlations, alongside comparative spectroscopic data from literature. The small coupling constant value (*J*_3,4_ = 3.0 Hz) indicated a *syn*-form for H-3 and H-4, whereas the larger coupling constant value (*J*_4,5_ = 8.0 Hz) suggested an *anti*-form for H-4 and H-5. Notably, the modified Mosher method was employed to confirm the absolute configurations at C-3 (Scheme S1, Supplementary Materials). Comparative analysis of selected ^1^H NMR chemical shifts for both MTPA esters established the C-3*S* configuration (Figures S1 and S22, Supplementary Materials). In contrast, the NOESY spectrum showed correlations of H-3 (*δ*_H_ 4.41) to H-4 (*δ*_H_ 5.18), confirming these protons have the same *β*-orientation. Indeed, the selective 1D NOE irradiation of H-4 showed a strong signal enhancement at H-3 and a minor effect at H-5, indicating the same configuration for H-3 and H-4. Conversely, irradiation of H-3 showed signal enhancement of H_2_-2 (*δ*_H_ 2.09 and 2.37) and H-4 (*δ*_H_ 5.18) without any effect on H-5. This observation suggested the H-3*β*, H-4*β*, and H-5*α* orientations. Therefore, the structure of compound **7** was fully established as a new compound and named rotundi B.

Our study demonstrated a useful tool and effective method to elucidate the structural configuration of quinic derivatives by applying the Mosher method and 1D selective NOE experiments for the first time. These methods accurately determined and resolved the complexity of H_2_-2 and H_2_-6 assignment in the quinic moiety.

### 3.3. Antioxidative Effect of AQAs

Previous study [14] demonstrated that CQA compounds exhibit patented radical scavenging activity. Therefore, the isolates were evaluated for their antioxidant activity using the DPPH assay. As a result, compounds **1**, **3**, and **8** displayed stronger radical scavenging activity (89.97%, 93.68%, and 85.98%, respectively) than those of the positive control (ascorbic acid, 79.31%) at a tested concentration of 100 µM. Compound **4** displayed a similar antioxidative effect compared to positive control. On the other hand, compounds **5**, **6**, and **9** exhibited significant scavenging activity to DPPH radicals with inhibition rates of 76.84%, 70.85%, and 72.27%, respectively, comparable to the positive control (79.31%). The remaining compounds (**2, 7**, and **10**) exhibited moderate to marginal antioxidative effects. At a concentration of 10 µM, compounds **1**–**4**, **6**, **7**, and **9** exhibited stronger inhibition on radical scavenging activity than those of ascorbic acid. Compounds **5**, **8**, and **10** showed a similar antioxidative effect to those of positive controls at 10 µM (Figure S23, Supplementary Materials).

### 3.4. AQAs Downstream Cytokine Production

**Figure 5:**
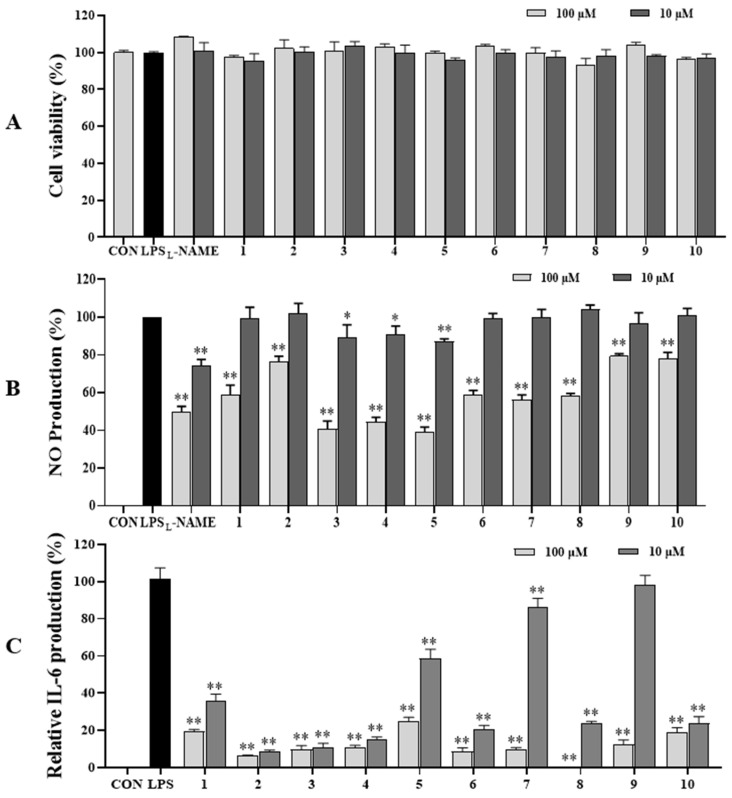
Cytotoxic (**A**), NO (**B**), IL-6 (**C**) production inhibitory effects of isolated compounds (**1**–**10** at 10 and 100 µM) in LPS-stimulated RAW264.7 cells. RAW264.7 cells were treated with compounds **1**–**10** (10 and 100 µM) for 1 h and stimulated with LPS (1 µg/mL) for 24 h. (**A**) The viability of cells was determined using an MTT assay. (**B**) The level of NO and IL-6 production in serum-free culture medium was measured. All experiments were performed in triplicates. The data are represented as mean ± SD. * *p* < 0.05, ** *p* < 0.001, compared to LPS-treated group.

The ability of the above isolates to inhibit NO and IL-6 production in LPS-stimulated RAW264.7 cells was evaluated. All compounds (**1**−**10**) demonstrated no effect on the cell viability of RAW264.7 cells under the conditions tested (Figure 5A). At a concentration of 100 µM, compounds **3**−**5** strongly reduced NO production, with NO production contents of 40.70%, 44.46%, and 39.05%, respectively, compared to those of non-sample-treated LPS (100%). Compounds **1** and **6**−**8** exhibited marked inhibition against NO production, with NO production found ranging from 56.12% to 58.88% compared to LPS stimulation (100%) without compound addition (Figure 5B). Other compounds showed minimal activity. At a concentration of 10 µM, compounds **3**–**5** significantly inhibited NO production induced by LPS-activated RAW264.7 cells. The positive control (L-NAME) displayed significant inhibition against NO production compared to cells treated with LPS at both concentrations of 10 µM and 100 µM.

In the IL-6 assay, all compounds effectively decreased IL-6 production. At a concentration of 100 µM, compound **8** dramatically reduced IL-6 production with an IL-6 production content of 0.20% compared to non-compound-treated cells with LPS stimulation (100%). Compounds **2**–**4**, **6**, **7**, and **9** substantially inhibited IL-6 production, with IL-6 production found ranging from 6.22% to 12.30% compared to those with LPS treatment (100%) without compound addition. Meanwhile, compounds **1**, **5**, and **10** also reduced IL-6 production, achieving an IL-6 production content ranging from 19.00% to 24.77%. At a concentration of 10 µM, compounds **1**–**4**, **6**, **8**, and **10** strongly suppressed IL-6 production induced by LPS-activated RAW264.7 cells compared to those of non-compound treatment with LPS. Furthermore, compounds **5** and **7** significantly reduced IL-6 production, compared to those with LPS treatment without compound addition (Figure 5C).

**Figure 6:**
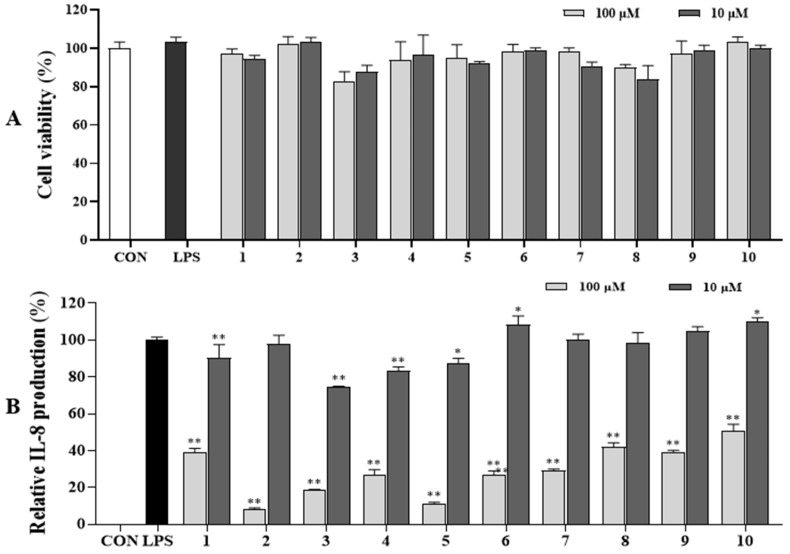
Cytotoxic (**A**) and IL-8 production inhibitory (**B**) effects of compounds in LPS-induced HT-29 cells. HT-29 cells were treated with compounds **1**–**10** (10 and 100 µM) for 2 h and stimulated with LPS (100 ng/mL) for 12 h. (**A**) The viability of cells was determined using an MTT assay. (**B**) The level of IL-8 in the culture media was measured with an ELISA kit. The values are expressed as mean ± standard deviation of three individual experiments. * *p* < 0.05, ** *p* < 0.01, compared to the LPS-treated group.

Furthermore, the isolated compounds **1**–**10** were assessed for their capability to inhibit IL-8 production in LPS-stimulated HT-29 cells. At a concentration of 100 µM, compounds **2** and **5** exhibited potent activity by markedly reducing IL-8 production with contents of 8.05% and 11.27%, respectively, compared to those of non-compound treatment (100%) with LPS-stimulated HT-29 cells. Compounds **3**–**7** also significantly decreased IL-8 production, with IL-8 production found ranging from 18.46% to 29.15%, compared to those of LPS-activated HT-29 cells without compound addition (100%). At a concentration of 10 µM, compounds **1** and **3**–**5** significantly suppressed IL-8 production induced by LPS-activated HT-29 without compound addition (Figure 6B). Compounds **8**–**10** effectively attenuated IL-8 production under the same testing conditions. All tested compounds did not cause any effect on HT-29 cell viability (Figure 6A).

To verify the inhibitory effect of active compounds, they were used as ligands in docking studies with the IL-8 protein (PDB ID 5D14); they formed ligand–protein complexes with strong docking scores, ranging from −8.2 to −6.9 kcal/mol. The ligand–protein interactions highlighted key residues within the binding pocket, such as ARG4, LYS9, GLU46, and CYS48, which are crucial for the ligand’s binding affinity when docked into the complex (Figure S24, Supplementary Materials).

### 3.5. Molecular Docking Guide AQAs Suppress iNOS and COX-2 Expression

A molecular docking study was performed to predict the binding affinity of the active compounds **3**–**5**, and **7** towards the iNOS protein (Figure S25, Supplementary Materials). Initially, the native ligand (AT2) of the protein was prepared using the MGL tool 1.5.7 and redocked into the protein to construct the complex with the aid of AutoDock4.0. The root means square deviation (RMSD) between the redocked ligand, and the crystallized ligand was found to be less than 2 Å, evidencing that the docking protocol successfully reproduced the binding mode of the crystallographic pose. When the active compounds **4**–**7** were docked into the inducible NO synthase (iNOS) protein (PDB ID 3E7G), they exhibited low binding energies of −5.59, −6.21, −7.21, and −6.55 kcal/mol, respectively, in comparison to the native ligand (AT2) of the 3E7G protein. Notably, the complex of compound **5** demonstrated the lowest docking score of −7.21 kcal/mol, surpassing that of the redocked crystallized ligand (−7.02 kcal/mol) due to its interaction and occupation of critical amino acids, including conventional hydrogen bonds with GLU377, ARG381, ASP382, TRP463, and TYR347. Conversely, the compound **7** complex also manifested a strong binding affinity with a docking score of −6.55 kcal/mol, interacting with key residues such as ARG199, THR376, GLU377, ARG381, ASP382, and VAL465. Similarly, the compound **4** complex revealed a robust binding affinity with a docking score of −6.21 kcal/mol through interactions with essential amino acids, namely TYR347, GLU377, VAL352, and TRP463 [15].

In the same manner, active compounds **3**–**5** and **7** were also docked to COX-2 protein (PDB ID 5IKQ). The docking protocol was also validated by redocking of the native ligand (JMS), indicating that the docking method is valid and feasible for designing molecules. Compounds **5** and **7** demonstrated low docking scores of −6.90 and −6.03 kcal/mol, respectively. These compounds showed good binding to protein due to interacting with key amino acids (TYR385 and SER530) and were similar to those of the redocked ligand. However, compounds **3** and **4** also displayed a binding energy of −4.21 and −3.52 kcal/mol, respectively (Figure S26, Supplementary Materials).

**Figure 7:**
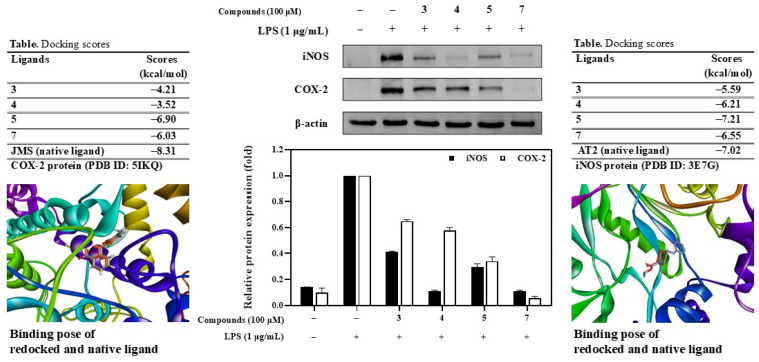
Effects of compounds **3**–**5**, and **7** on iNOS and COX-2 expression induced by LPS on RAW264.7 cells. Relative density was calculated as the ratio of the expression level of each protein with β-actin. Docking scores of compounds **3**–**5**, and **7** and native ligand (AT2, red) into iNOS protein (PDB ID 3E7G). Docking scores of compounds **3**–**5**, and **7** and native ligand (JMS, red orange) into COX-2 protein (PDB ID 5IKQ).

The inducible enzymes iNOS and COX-2 were found to be upregulated by LPS-stimulated RAW264.7 cells. Upon treatment with the active compounds **3**–**5**, and **7**, a significant decrease in the expression levels of these enzymatic proteins was observed, with levels lower than those seen in cells treated with LPS alone. Notably, these compounds did not affect the expression of β-actin. Specifically, compounds **4** and **7** more effectively downregulated iNOS expression than compounds **3** and **5**. In contrast, compound **7** exhibited the most substantial reduction in COX-2 expression when compared to compounds **3**–**5** (Figure 7). These findings imply that the active compounds may inhibit the inflammatory signaling pathway.

### 3.6. Compounds ***2***–***5***, ***7***, and ***8*** Inhibited LPS-Induced p38 MAPK Phosphorylation in RAW264.7 Macrophages

When exploring the strong active compounds **2**–**5**, **7**, and **8** for their effects on p38 MAPK protein through in silico docking to predict their binding affinity, compound **8** demonstrated a low docking score of −6.71 kcal/mol, indicating interactions between compound **8** and the receptor, involving amino acids such as LYS53, HIS107, MET109, and TYR35. Compounds **2**–**5** and **7** also exhibited low binding energies ranging from −6.69 to −5.09 kcal/mol, suggesting the feasibility of conducting a p38 MAPK pathway assay (Figure S27, Supplementary Materials).

**Figure 8:**
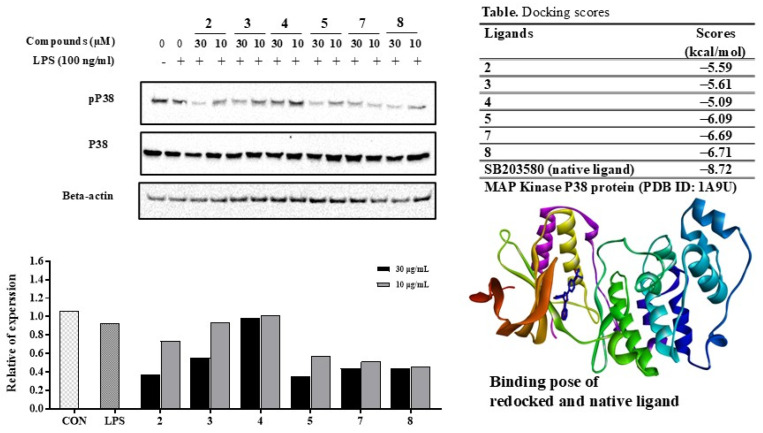
Effects of compounds **2**–**5**, **7**, and **8** in LPS-induced p38 MAPK signaling pathway in RAW264.7 cells. Relative density was calculated as the ratio of the expression level of each protein with β-actin. Docking scores of compounds **2**–**5**, **7**, and **8** and native ligand (SB203580, dark blue) into p38 MAPK protein (PDB ID 1A9U) in LPS-induced p38 MAPK signaling pathway in RAW264.7 cells. Relative density was calculated as the ratio of the expression level of each protein with β-actin. Docking scores of compounds **2**–**5**, **7**, and **8** and native ligand (SB203580) into p38 MAPK protein (PDB ID 1A9U).

Subsequently, the effects of these active constituents on the mitogen-activated protein kinase (MAPK) signaling pathway were investigated through the phosphorylation of MAPK pathway proteins, such as p38 kinase (p38). As depicted in Figure 8, compounds **2**–**5**, **7**, and **8** effectively reduced p38 phosphorylation.

## 4. Discussion

Inflammation is a bodily protective response against stimuli such as infections that aims to eradicate pathogens while facilitating tissue repair and recovery [22]. Characterized by symptoms including swelling, redness, pain, and a loss of tissue function, inflammation is central to the pathogenesis of chronic diseases such as bowel diseases, arthritis, cardiovascular disease, and cancer, involving inflammatory cytokines and mediators [23]. AQAs offer significant improvements in health benefits and have been deemed safe for therapeutic pathways in the prevention and treatment of chronic and cardiovascular diseases [24]. This is attributed to their structural properties that endow them with a wide range of biological properties, including antibacterial, antiparasitic, neuroprotective, anti-inflammatory [25], anticancer, antiviral [18], and antidiabetic effects [26]. Given the novel bioactivities of AQAs, the exploration of these compounds has garnered widespread interest from the global scientific community. In our research, we isolated and identified two novel AQAs (**5** and **7**) and eight known compounds from *V. rotundifolia* by using the supercritical fluid extraction method employed for the first time for this plant. The structural elucidation of the isolated compounds was achieved through NMR and high-resolution mass spectrometry, identifying all isolates as quinic acid derivatives. Notably, this study reports the isolation and identification of 3,4-di-*O*-caffeoylquinic acid, 3,4-di-*O*-caffeoylquinic acid methyl ester, 4,5-di-*O*-caffeoylquinic acid, 4,5-di-*O*-caffeoylquinic acid methyl ester, and 5-*O*-caffeoylquinic acid methyl ester from *V. rotundifolia* for the first time. The absolute structures of these compounds were confirmed using 1D-NOE analysis and chemical methods, including decoupling with modified Mosher and PGME methods. The 1D-NOE spectrum analysis enabled precise assignments of H_2_-2 and H_2_-6, facilitating the determination of H-3, H-4, and H-5 positions via selective irradiation. The absolute configurations of the quinic acid moieties in compounds **5** and **7** were determined using a modified Mosher method, while hydrolysis followed by the PGME method identified the configuration at C-1. Our study introduces simple and effective techniques for determining chiral centers in quinic acid derivatives, potentially aiding other researchers in the absolute determination of H_2_-2 and H_2_-6.

To identify compounds with anti-inflammatory properties, we utilized the NO assay to evaluate their ability to suppress NO production in LPS-induced RAW264.7 cells. Compounds **3**–**5** demonstrated significant inhibition of NO production. In addition, new compound **7** also moderately inhibited NO production. A structure–activity relationship (SAR) revealed that compounds **1**, **3**, **4**, **6**, and **8**, being di-*O*-caffeoyl quinic acids, displayed varied anti-inflammatory effects, with compounds **3** and **4** showing stronger effects than **1**, **6**, and **8**. This suggested the importance of the 3,5-di-*O*-caffeoyl functional groups for anti-inflammatory activity. Between compounds **5** and **7**, both benzoyl-5-caffeoyl quinic acids, compound **5** exhibited a more potent inhibitory effect, indicating the 3-O-benzoyl functional group’s significance in the NO assay. Overall, di-*O*-hydroxycinnamic quinic acids demonstrated superior anti-inflammatory properties compared to mono-hydroxycinnamic quinic acids.

NO and PGE_2_, critical mediators in inflammation, are produced by inducible NO synthase (iNOS) and cyclooxygenase type 2 (COX-2), respectively. Inhibiting these mediators can provide therapeutic benefits for inflammatory diseases [27]. The active compound **4** notably reduced iNOS levels, while **7** downregulated both iNOS and COX-2 expression in a dose-dependent manner.

IL-6 is a cytokine involved in trans-signaling mechanisms that activate pro-inflammatory pathways [28], whereas IL-8 is a chemokine produced by immune cells under inflammatory conditions [29]. The compounds significantly inhibited IL-6 production in LPS-induced RAW264.7 cells, particularly compound **8**, which nearly completely inhibited IL-6 production at 100 µM. Other compounds were found to show an inhibitory effect on IL production of approximately 6.22% to 24.77%, compared to controls. Notably, all compounds were non-toxic to the cells.

In the IL-8 assay, compounds **2** and **5** notably suppressed IL-8 production in LPS-stimulated HT-29 cells, with others showing strong to moderate inhibition in a dose-dependent manner, without exhibiting cytotoxicity. The SAR indicated the 3-hydroxynamic acid moiety’s role in suppressing IL-8 production, while hydroxynamic acids at C-4 were less effective. Among compounds **5** and **7**, both benzoyl-caffeoyl quinic derivatives, **5** showed stronger inhibition, underscoring the 3-*O*-benzoyl group role in modulating IL-8 production.

The activation of mitogen-activated protein kinase (MAPK) pathways, including extracellular signal-regulated kinase, c-Jun N-terminal kinases, and p38 MAPK, is a key event in inflammatory responses [30,31]. The studied compounds **2**–**5**, **7**, and **8** reduced p38 phosphorylation, demonstrating their potential in modulating inflammatory responses through MAPK signaling.

An in silico approach was employed to facilitate the bioassay. Docking studies revealed that the compounds not only significantly inhibited NO production but also demonstrated high binding affinity to iNOS and COX-2 proteins, as evidenced by low docking scores. This can be attributed to the compounds occupying the same region as the native ligand of the protein, potentially interfering with the key amino acids within the protein’s binding pockets. The docking results further indicated low docking scores when active compounds **2**–**5**, **7**, and **8** formed complexes with the p38 MAPK protein, underscoring their binding affinity towards the respective protein. These docking results provide supportive evidence for guided experimentation.

Free radical production, resulting from an imbalance of natural oxidants, leads to inflammation-associated diseases. Consequently, the isolates **1**–**10** were evaluated for their antioxidant capacities. These compounds demonstrated significant antioxidant effects, as shown by their DPPH radical scavenging activities at the tested concentrations. SAR was derived from the observed radical scavenging activities. Compounds **1** and **2** were identified as 3,4-di-*O*-caffeoylquinic acids, with compound **1** exhibiting a markedly stronger effect than **2**. Compounds **3**–**5** were classified as 3,5-di-*O*-caffeoylquinic acids, among which compound **3** showed the highest radical scavenging activity, followed by **4** and **5**. Therefore, the presence of the caffeoyl substituent is crucial for DPPH radical scavenging activity. However, the involvement of compounds **7** and **8** suggested an opposite effect due to the presence of *p*-benzoyl moiety substitution, contrasting with the favorable influence of the caffeoyl substituent on the radical scavenging activity of 4,5-di-*O*-caffeoylquinic acids. The antioxidative potential of these compounds can be elucidated by their chemical structure characteristics. In summary, AQAs may donate hydrogen atoms to neutralize free radicals or inhibit oxidation reactions. Upon donating hydrogen atoms, AQAs are converted into their respective phenoxyl radicals, which are rapidly stabilized through resonance stabilization [32]. Consequently, the reaction products diminish the oxidative capability of free radicals.

## 5. Conclusions

In conclusion, our study unveiled the chemical composition and structural details of ten AQAs from *V. rotundifolia* using the SFE technique. Among these, two were new and eight were previously known compounds, all distinguished through the inaugural application of 1D-NOE spectra alongside comprehensive spectroscopic data analysis. Compounds **1**, **3**, **4**, **6**, **8**, and **9** exhibited potent antioxidative properties via DPPH radical scavenging activity. Furthermore, compounds **3**–**5**, **7**, **2**–**4**, and **6**–**9** markedly reduced NO and IL-6 production induced by LPS-activated RAW264.7 cells. Compounds **2**–**7** suppressed IL-8 production induced by LPS-stimulated HT-29 cells. These active compounds (**3**–**5**, and **7**) also reduced the expression levels of iNOS and COX-2 proteins. Additionally, the molecular mechanism underlying the anti-inflammatory activity of compounds **2**–**5**, **7**, and **8,** was ascribed to the inhibition of MAPK signaling, specifically through the prevention of p38 phosphorylation. Docking studies corroborated the binding affinity of these compounds to proteins (IL-8, iNOS, COX-2, and p38 MAPK), revealing interactions with key residues within the binding pockets of their respective complexes. Our findings approved the traditional use towards inflammatory diseases with specific evidence. Collectively, our findings suggest that compounds **3**–**5**, and **7** hold promise as potential therapeutic agents for the prevention and treatment of inflammatory diseases in future endeavors. This study offers guidance for future research and the creation of enriched acyl-quinic acids targeting functional goods that support natural sources of health benefits.

## Figures and Tables

**Table 1:** ^1^H (400 MHz) and ^13^C NMR (100 MHz) spectroscopic data for compounds **5** and **7**, acquired in methanol-*d*_4_. *δ*_H_, multiplicity (*J* in Hz).

No.	5		7	
	*δ*_C_	*δ*_H_	*δ*_C_	*δ*_H_
1	74.8	–	75.8	-
2	37.0	2.18 (1H, dd, 7.6, 13.7) 2.35 (1H, dd, 4.1, 13.7)	38.6	2.09 (1H, dd, 6.9, 14.1) 2.37 (1H, dd, 3.7, 14.1)
3	72.3	5.48 (1H, dt, 3.7, 7.3)	68.5	4.41 (1H, m)
4	70.1	4.00 (1H, dd, 3.2, 6.9)	75.2	5.18 (1H, dd, 3.1, 8.0)
5	72.1	5.35 (1H, ddd, 4.2, 7.1, 11.1)	69.0	5.60 (1H, m)
6	35.8	2.20 (2H, m)	38.5	2.23 (1H, dd, 4.6, 13.7) 2.30 (1H, d, 8.4, 4.9)
7	175.8	–	175.2	-
7-*O*CH_3_	53.0	3.68 (3H, s)	53.1	3.71 (3H, s)
BA				
1′	122.6	–	123.1	-
2′	133.2	7.96 (2H, d, 8.7)	133.1	7.92 (2H, d, 8.8)
3′	116.0	6.82 (2H, d, 8.7)	116.3	6.81 (2H, d, 8.8)
4′	163.5	–	164.1	-
5′	116.0	6.82 (2H, d, 8.7)	116.3	6.81 (2H, d, 8.8)
6′	133.2	7.96 (2H, d, 8.7)	133.1	7.92 (2H, d, 8.8)
7′	167.7	–	167.5	
CFA				
1″	127.6		127.3	-
2″	115.1	7.04 (1H, d, 2.3)	115.0	6.99 (1H, d, 1.0)
3″	146.9	–	146.9	-
4″	149.8	–	150.1	-
5″	116.6	6.77 (1H, d, 8.2)	116.5	6.76 (1H, d, 8.2)
6″	123.1	6.96 (1H, dd, 2.3, 8.2)	121.7	6.91 (1H, dd, 1.0, 8.2)
7″	114.9	7.54 (1H, d, 16.0)	114.3	7.48 (1H, d, 16.0)
8″	147.4	6.22 (1H, d, 16.0)	147.7	6.14 (1H, d, 16.0)
9″	168.1	–	167.9	-

## Data Availability

All data is contained within the article.

## Supplementary Materials

The following supporting information can be downloaded at: https://www.mdpi.com/article/10.3390/antiox13101235/s1. SchemeS1: Determination of absolute configurations of **5** and **7** by chemical derivatization analysis using modified Mosher method; Figure S1: Δ*δ*_H_ values (in ppm) = *δ*_H,S_ − *δ*_H,R_ obtained for (*S*)- and (*R*)-MTPA esters (**5a**/**7a** and **5b**/**7b**); Scheme S2: Determination of absolute configuration of quinic acid moiety by PGME method; Figure S2: HR-ESI-MS spectrum of compound **5**; Figures S3: NMR spectra of compound **5**; Figure S10: Low mass spectrometry of MTPA ester of compound **5**; Figure S11: ^1^H NMR spectra of *S*- and *R*-MTPA derivatives (**5a** and **5b**); Figure S12: Low mass spectrometry of PGME ester of compound **5**; Figure S13: ^1^H NMR spectra of *S*- and *R*-PGME amide derivatives (**5c** and **5d**); Figure S14: HR-ESI-MS spectrum of compound **7**; Figures S15–21: NMR spectra of compound **7**; Figure S22: ^1^H NMR spectra of *S*- and *R*-MTPA derivatives (**7a** and **7b**); Figure S23: Antioxidant effect of compounds (**1–10**); Figure S24: Interactions of **2** (blue), **3** (yellow), **4** (pink), **5** (teal), **6** (grey), **7** (orange) docked into IL-8 protein (PDB ID: 5D14) with 3D visualization; Figure S25: Interactions of **3** (yellow), **4** (pink), **5** (teal), **7** (lightblue), and AT2 (brown) docked into iNOS protein (PDB ID: 3E7G) with 3D visualization; Figure S26: Interactions of **3** (yellow), **4** (pink), **5** (teal), **7** (lightblue), and JMS (orange) docked into COX-2 protein (PDB ID: 5IKQ) with 3D visualization; Figure S27: Interactions of **2** (magenta), **3** (yellow), **4** (pink), **5** (cyan). **7** (violet), **8** (grey), and SB203580 (blue) docked into MAP Kinase P38 protein (PDB ID: 1A9U) with 3D visualization.
