# Supplementary material for: Bioactivities of Quinic Acids from Vitex rotundifolia Obtained by Supercritical Fluid Extraction
Source: Antioxidants (Basel). 2024 Oct 14;13(10):1235. doi: 10.3390/antiox13101235 (PMC11504455; doi:10.3390/antiox13101235)
Supplement: Supplementary file 1 [file antioxidants-13-01235-s001.zip › antioxidants-3182656-supplementary.pdf]

Supplementary materials

Bioactivities of Quinic Acids from Vitex rotundifolia Obtained by Supercritical Fluid Extraction

Duc Dat Le[1,2†], Young Su Jang[2,†], Vinhquang Truong[1], Soojung Yu[3], Thientam Dinh[1] and Mina Lee[a,c,*]

[1]College of Pharmacy and Research Institute of Life and Pharmaceutical Sciences, Sunchon National University, 255 Jungangno, Suncheon 57922, Jeonnam, Republic of Korea

[2]Nano Bio Research Center, Jeonnam Bio Foundation, Jangseong 57248, Jeonnam, Republic of Korea

[3]Department of Natural Cosmetics Science, Graduate School, Sunchon National University, 255 Jungangno, Suncheon 57922, Jeonnam, Republic of Korea.

[†]These authors contributed equally to this work.

*Correspondence: minalee@sunchon.ac.kr/ minalee@scnu.ac.kr; Tel.: +82-61-750-3764; Fax: +82-61-750-3708

Table of Contents

1.Experiments 1

1.1. Separation and isolation.......... 1

1.2. Modified Mosher assay 3

Scheme S1. Determination of absolute configurations of **5** and **7** by chemical derivatization analysis using modified Mosher method 3

Figure S1. $\Delta\delta_H$ values (in ppm) = $\delta_{H,S} - \delta_{H,R}$ obtained for (*S*)- and *(R)*-MTPA esters (**5a**/**7a** and **5b**/**7b**) 4

1.3. PGME assay 4

Scheme S2. Determination of absolute configuration of quinic acid moiety by PGME method 5

1.4. Spectroscopic data of compounds (5 and 7): 6

Figure S2. HR-ESI-MS spectrum of compound **5** 6

Figure S3. ^{1}H-NMR (400 MHz, CD_3OD) spectrum of compound **5** 6

Figure S4. ^{13}C-NMR (100 MHz, CD_3OD) spectrum of compound **5** 7

Figure S5. ^{1}H-^{1}H COSY spectrum of compound **5** 7

Figure S6. ^{1}H-^{13}C HMQC spectrum of compound **5** 8

Figure S7. ^{1}H-^{13}C HMBC spectrum of compound **5** 8

Figure S8. ^{1}H-^{1}H NOESY spectrum of compound **5** 9

Figure S9. Selective 1D NOE spectrum of **5** 9

Figure S10. Low mass spectrometry of MTPA ester of compound **5** 10

Figure S11. ^{1}H NMR spectra of *S*- and *R*-MTPA derivatives (**5a** and **5b**) 10

Figure S12. Low mass spectrometry of PGME ester of compound **5** 11

Figure S13. ^{1}H NMR spectra of *S*- and *R*-PGME amide derivatives (**5c** and **5d**).......... 11

Figure S14. HR-ESI-MS spectrum of compound **7** 10

Figure S15. ^{1}H-NMR (400 MHz, CD_3OD) spectrum of compound **7** 10

Figure S16. ^{13}C-NMR (100 MHz, CD_3OD) spectrum of compound **7** 11

Figure S17. ^{1}H-^{1}H COSY spectrum of compound **7** 11

Figure S18. ^{1}H-^{13}C HMQC spectrum of compound **7** 12

Figure S19. ^{1}H-^{13}C HMBC spectrum of compound **7** 12

Figure S20. ^{1}H-^{1}H NOESY spectrum of compound **7** 13

Figure S21. Selective 1D NOE spectrum of **7** 13

Figure S22. ^{1}H NMR spectra of *S*- and *R*-MTPA derivatives (**7a** and **7b**)....................................15
Figure S23. Antioxidant effect of compounds (**1–10**) 16
Figure S24. Interactions of **2** (blue), **3** (yellow), **4** (pink), **5** (teal), **6** (grey), **7** (orange) docked into IL-8 protein (PDB ID: 5D14) with 3D visualization 17
Figure S25. Interactions of **3** (yellow), **4** (pink), **5** (teal), **7** (lightblue), and AT2 (brown) docked into iNOS protein (PDB ID: 3E7G) with 3D visualization 17
Figure S26. Interactions of **3** (yellow), **4** (pink), **5** (teal), **7** (lightblue), and JMS (orange) docked into COX-2 protein (PDB ID: 5IKQ) with 3D visualization..18
Figure S27. Interactions of **2** (magenta), **3** (yellow), **4** (pink), **5** (cyan). **7** (violet), **8** (grey), and SB203580 (blue) docked into MAPK P38 protein (PDB ID: 1A9U) with 3D visualization...........18

1. Experiments

1.1. Separation and isolation

The EtOAc fraction (184.69 g) was subjected to an open-column chromatography over silica gel using multiple step gradients consisting of methylene chloride (MC):methanol (M):distilled water (DW) (15:1:0.001 to 1:2:0.1, each 2 L) to obtain ten sub-fractions (E1–E10). Then, subfraction E3 (10.0 g) was seperated using a flash column over ODS silica gel, eluted with acetonitrile in distilled water with ration ranging 1/5 to 5/1 (each 0.3 L) to obtain 26 sub-fractions (E3A–E3Z). Subfraction E3H was isolated by using MPLC (YMC Triart ODS C_{18}, 5 µm, 250 x 20 mm, Tokyo, Japan) detect by UV at 254 nm, flow rate 5.0 mL/min, eluted by a solvent system including water (A, containing 0.1% formic acid) and ACN (B) as follow: 0 min (22% B)-60 min (33% B)-110 min (100% B) to obtain compounds **7** (t_R 41 min) and **8** (t_R 45 min). Subfraction E3I was also isolated by a RP-MPLC (YMC Triart ODS C_{18}, 5 µm, 250 x 20 mm, Tokyo, Japan), UV detection at 254 nm, flow rate 5.0 mL/min, eluting solvent system including water (A, containing 0.1% formic acid) and ACN (B) as follow: 0 min (26% B)-65 min (24% B)-110 min (40% B)-140 min (100% B) to obtain subfraction E3I-39-50, and further purified by using a isocratic solvent system with 70% acetonitrile during 40 mintutes to obtain compound **4** (t_R 18 min) and compound **5** (t_R 19 min). Subfraction E3N was isolated to prep HPLC using a column (YMC Triart ODS C_{18}, 5 µm, 250 x 20mm, Tokyo, Japan) with UV detection at wavelength 254 nm and 300 nm, flow rate 3.0 mL/min, eluting with a mobile phase of water (containing 0.3% formic acid, A) and acetonitrile (B) as a gradient solvent system [0 min (23% B)-40 min (30% B)-60 min (100% B)] to obtain compounds **2** (t_R 22 min). Subfraction E4 was continually seperated using open-column chromatography over ODS silica gel using gradient solvent system consisting of acetonitrile and distilled water to obtain 26

sub-fractions (E4A–E4Z). Sub-fraction E4B was loaded onto the above separation condition using a gradient solvent system from 0 min (14% B) to 100 min (100% B); a wavelength of 202 and 254 nm was used to yield compounds **6** (t_R 12 min). Subfraction E4F was subjected to a RP-MPLC with a column (YMC Triart ODS C_{18}, 5 µm, 250 x 20 mm, Tokyo, Japan) eluting with a gradient solvent system of acetonitrile in water (containing 0.1% formic acid) from 25% to 100% for 100 min; flow rate of 5.0 mL/min; UV detection at 202 and 254 nm; to obtain compound **3** (t_R 20 min). The Bu fraction (75.8 g) was subjected to an open-column chromatography over silica gel using multiple step gradients consisting of MC:M:DW to obtain eight sub-fractions (B1–B8). Subfraction B7 was subjected to a RP-MPLC (YMC Piscopack A7) eluting with solvent system including water (A, containing 0.1% formic acid) and ACN (B) as follow: 0 min (10% B) to 70 min (100% B); flow rate of 5.0 mL/min; UV detection at 203, 262 and 310 nm; to obtain nine subfractions (B7A–B7I). Subfraction B7F was further purified on HPLC chromatographed over ODS column (YMC Triart ODS C_{18}, 5 µm, 250 x 20 mm, Tokyo, Japan) using isocratic solvent system consisting water: acetonitrile (80% : 20%) to obtain three subfractions (B7F0–B7F2). Subfraction B7F1was isolated to prep HPLC using Triart ODS C_{18} column (10 × 250 mm, 5 µm, YMC, Tokyo, Japan), UV detection at wavelength 254 nm, flow rate 3.0 mL/min, eluting with isocratic solvent system consisting water: acetonitrile (81% : 19%) in 80 mintutes to afford compound **1** (t_R 67 min). Subfraction B7C was further purified on a preparative high-performance liquid chromatography (prep HPLC) using Triart ODS C_{18} column (10 × 250 mm, 5 µm, YMC, Tokyo, Japan), UV detection at wavelength 254 nm, flow rate 3.0 mL/min, eluting with a mobile phase of water (containing 0.1% formic acid, A) and acetonitrile (B) as a gradient solvent system from 0 min (12%) to 80 min (100%) to obtain two compounds **9** (t_R 39 min) and **10** (t_R 17.8 min).

*1.1.1. Compounds **5** and **7** characterizations*

Rotundi A (**5**): Pale yellowish powder; $[\alpha]_D^{23}$-130 (c 0.026, MeOH); UV (MeOH) λ_{max} 193 (2.5), 252 (1.6) 328 (1.1) nm; ^{1}H NMR (400 MHz, CD_3OD) and ^{13}C NMR (100 MHz, CD_3OD) data see Tables 1; HR-ESI-MS m/z 487.1246 $[M–H]^-$, calcd. for $C_{24}H_{23}O_{11}$, 487.1240, 349.0930 $[M–H–benzoyl]^-$, 161.0246 $[Caffeoyl]^-$, 137.0246 $[Benzoyl]^-$.

Rotundi B (**7**): Pale yellowish powder; $[\alpha]_D^{23}$-20 (c 0.018, MeOH); UV (MeOH) λ_{max} 194 (2.4), 252 (1.4) 328 (1.0) nm; ^{1}H NMR (400 MHz, CD_3OD) and ^{13}C NMR (100 MHz, CD_3OD) data see Tables 1; HR-ESI-MS m/z 487.1244 [M+Na]+, calcd. for $C_{24}H_{23}O_{11}$, 487.1240, 349.0929 $[M–H–benzoyl]^-$, 161.0246 $[Caffeoyl]^-$, 137.0246 $[Benzoyl]^-$.

1.2. Modified Mosher assay:

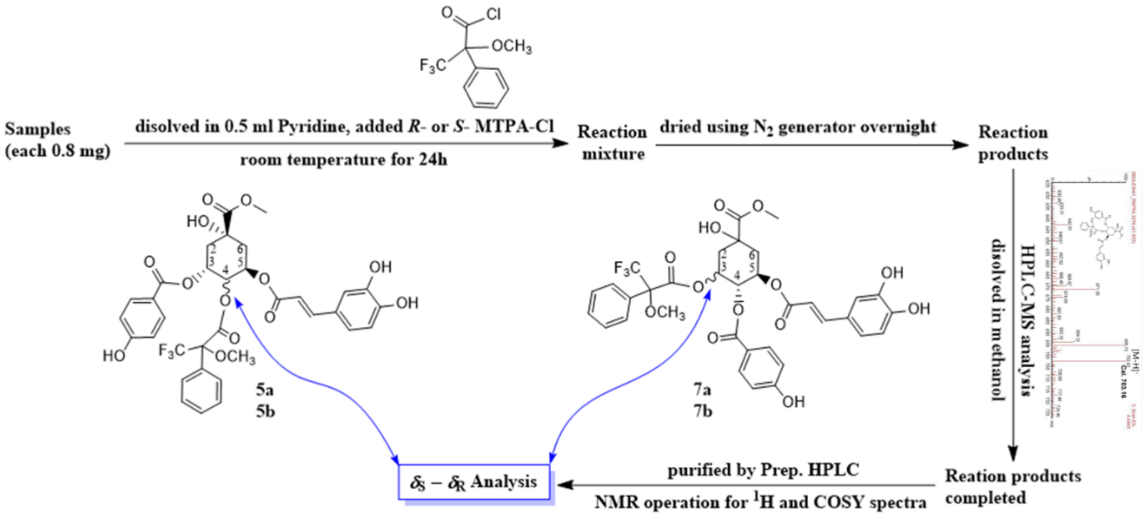

Scheme S1. Determination of absolute configurations of **5** and **7** by chemical derivatization analysis using modified Mosher method.

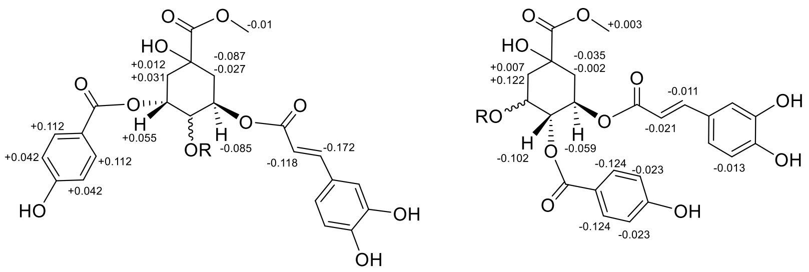

5a/5b **7a/7b**

Figure S1. $\Delta\delta_H$ values (in ppm) = $\delta_{H,S} - \delta_{H,R}$ obtained for (*S*)- and *(R)*-MTPA esters (**5a**/**7a** and **5b**/**7b**)

1.2.1. Spectroscopic data of MTPA diester derivatives

S-MTPA-ester (**5a**): ^{1}H NMR (400 MHz, CD_3OD): 2.298 (2H, m, H_2-6), 2.305 (1H, m, H-2a), 2.502 (1H, dd, 3.3, 14.4, H-2b), 3.719 (3H, s, CH_3-7), 5.566 (1H, dd, 3.3, 9.1, H-4), 5.742 (1H, m, H-5), 5.843 (1H, m, H-3), 6.401 (1H, d, 16.0, H-8''), 7.605 (1H, d, 16.0, H-7''), 7.154 (2H, overlap, H-2''/H-6''), 7.385 (1H, d, 8.2, H-5''), 7.603 (1H, d, 16.0, H-7''), 7.305 (2H, d, 8.5, H-3'/5'), 8.145 (2H, d, 8.5, H-2'/6').

R-MTPA-ester (**5b**): ^{1}H NMR (400 MHz, CD_3OD): 2.293 (1H, m, H-2a), 2.325 (1H, m, H-6a), 2.385 (1H, m, H-6b), 2.471 (1H, dd, 2.9, 14.7, H-2b), 3.729 (3H, s, CH_3-7), 5.584 (1H, dd, 3.6, 9.3, H-4), 5.788 (1H, m, H-3), 5.827 (1H, m, H-5), 6.573 (1H, d, 15.7, H-8''), 7.151 (1H, brs, H-2''), 7.721 (1H, d, 15.7, H-7''), 7.263 (2H, d, 8.8, H-3'/5'), 8.033 (2H, d, 8.5, H-2'/6').

S-MTPA-ester (**7a**): ^{1}H NMR (400 MHz, CD_3OD): 2.290 (1H, dd, 6.4, 13.9, H-6a), 2.371 (overlap, H-2a), 2.383 (overlap, H-6b), 2.583 (1H, d, 2.7, 15.2, H-2b), 3.682 (3H, s, 7-OCH_3), 5.421 (1H, dd, 3.0, 8.0, H-4), 5.727 (1H, m, H-5), 5.807 (1H, m, H-3), 6.485 (1H, d, 16.0, H-8''), 7.186 (overlap, H-3'/5'), 7.367 (1H, d, 8.5, H-5'') 7.629 (overlap, H-6''), 7.639 (overlap, H-7''), 7.868 (2H, d, 8.8, H-2'/6').

R-MTPA-ester (**7b**): ^{1}H NMR (400 MHz, CD_3OD): 2.248 (1H, dd, 6.2, 14.4, H-2a), 2.291(overlap, H-6a), 2.419 (1H, dd, 9.1, 13.7, H-6b), 2.575 (1H, d, 3.5, 14.4, H-2b), 3.679 (3H, s, 7-OCH_3), 5.525 (1H, dd, 3.7, 8.2, H-4), 5.786 (1H, m, H-5), 5.830 (1H, m,

H-3), 6.506 (1H, d, 16.0, H-8''), 7.646 (overlap, H-6''), 7.650 (1H, d, 16.0, H-7''), 7.992 (2H, d, 8.8, H-2'/6'), 7.209 (2H, d, 8.8, H-3'/5'), 7.380 (1H, d, 8.2, H-5'').

1.3. PGME assay

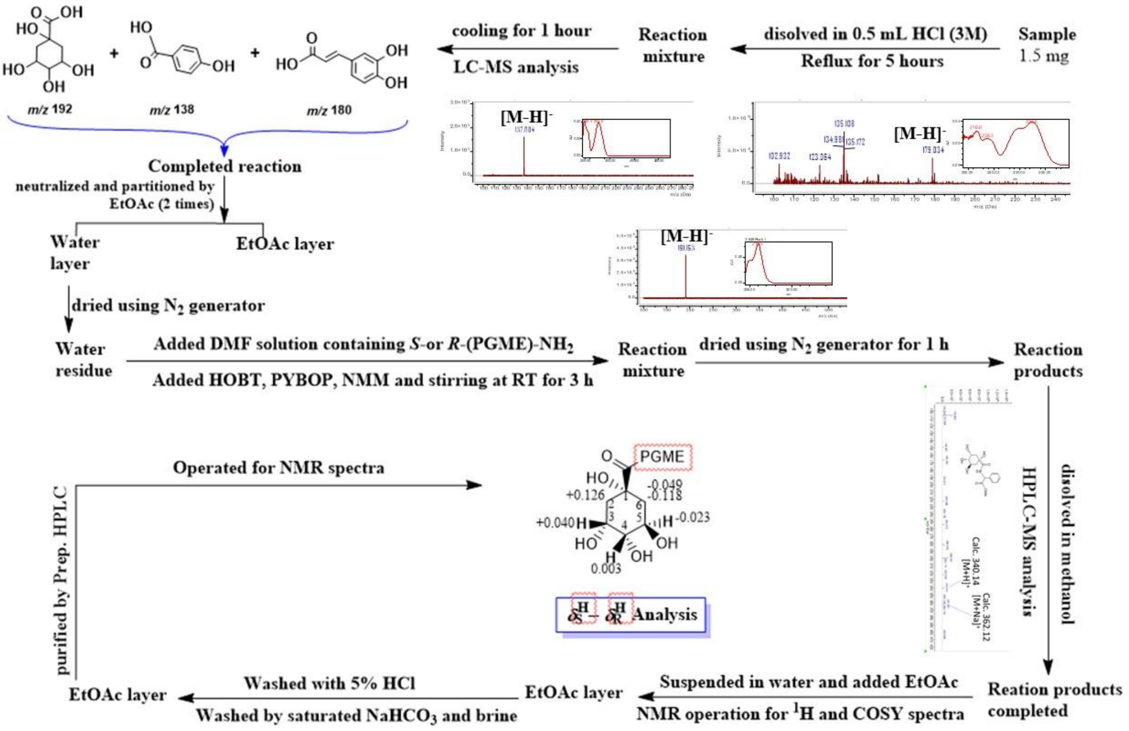

Scheme S2. Procedure of hydrolysis of compound **5** followed by application of PGME method to determine the configuration of C-1.

1.3.1. Spectroscopic data of PGME diester derivatives

S-PGME-amide (**5c**): ^{1}H NMR (400 MHz, CD_3OD): 1.834 (1H, dd, 10.9, 13.3, H-6a), 1.934 (1H, dd, 5.3, 13.3, H-6b), 1.995 (2H, m, H_2-2), 3.379 (1H, dd, 3.2, 9.2, H-4), 3.720 (PGME-OCH_3), 3.990 (1H, ddd, 4.8, 8.9, 10.9, H-5), 4.153 (1H, q, 3.2, H-3), 7.339-7.386 (5H, PGME-aryl).

R-MTPA-amide (**5d**): ^{1}H NMR (400 MHz, CD_3OD): 1.869 (2H, m, H_2-2), 1.883 (1H, m, H-6a), 2.052 (1H, dd, 4.7, 13.2, H-6b), 3.376 (1H, dd, 3.0, 8.9, H-4), 3.718 (PGME-OCH_3), 4.013 (1H, m, H-5), 4.113 (1H, q, 3.8, H-3), 7.337-7.386 (5H, aryl).

*1.4. Spectroscopic data of compounds (**5** and **7**):*

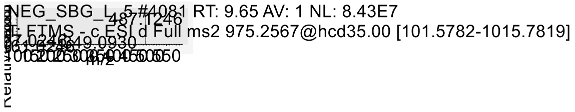

Figure S2. High mass spectrometry of **5**

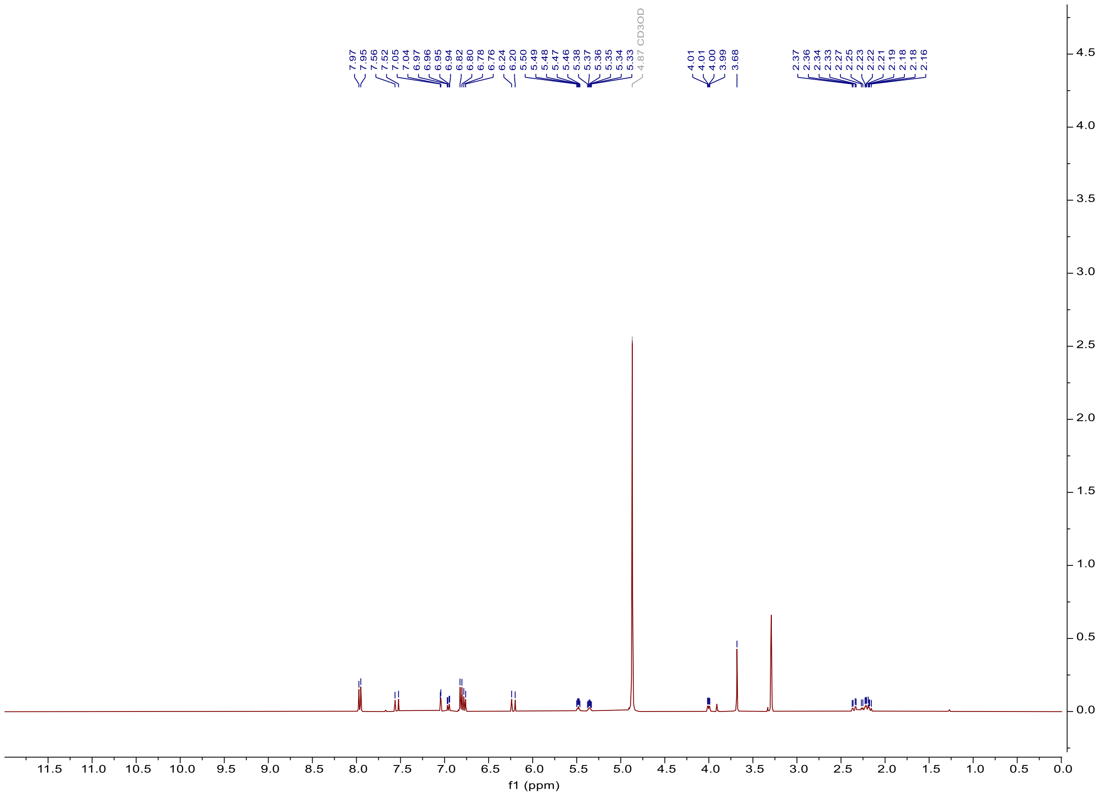

Figure S3. ^{1}H-NMR (400 MHz, CD_3OD) spectrum of **5**

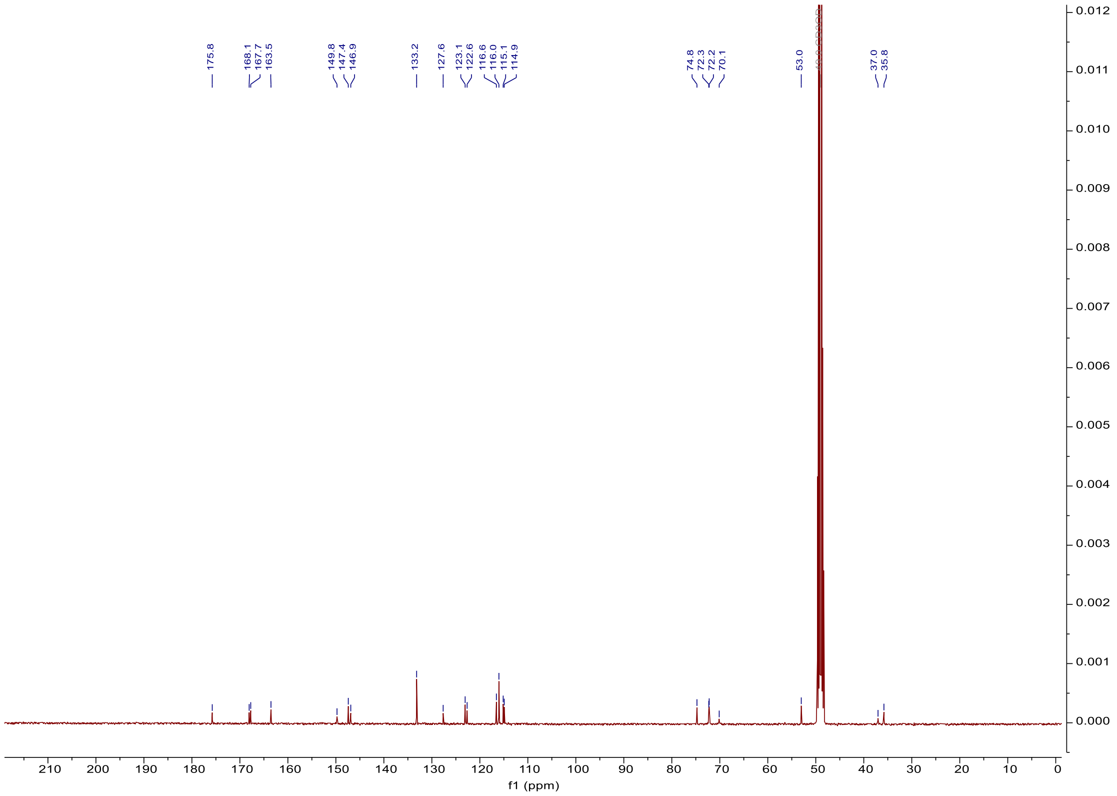

Figure S4. ^{13}C-NMR (100 MHz, CD_3OD) spectrum of **5**

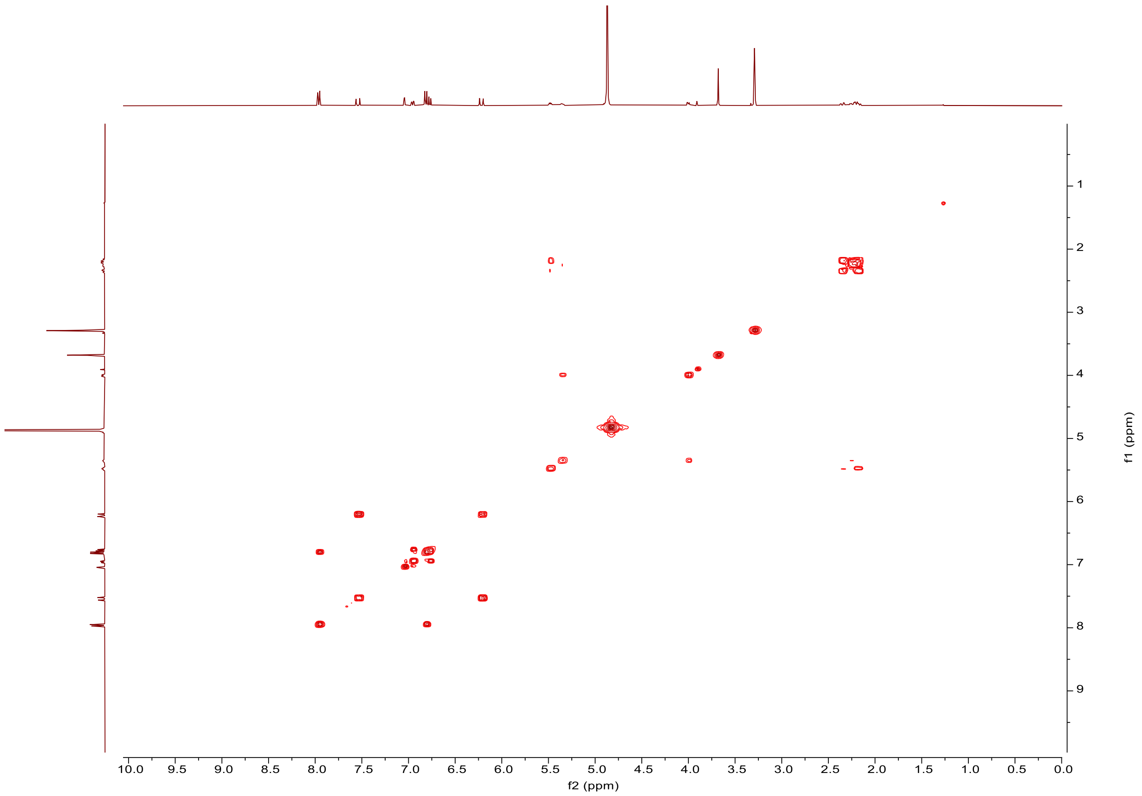

Figure S5. COSY spectrum of **5**

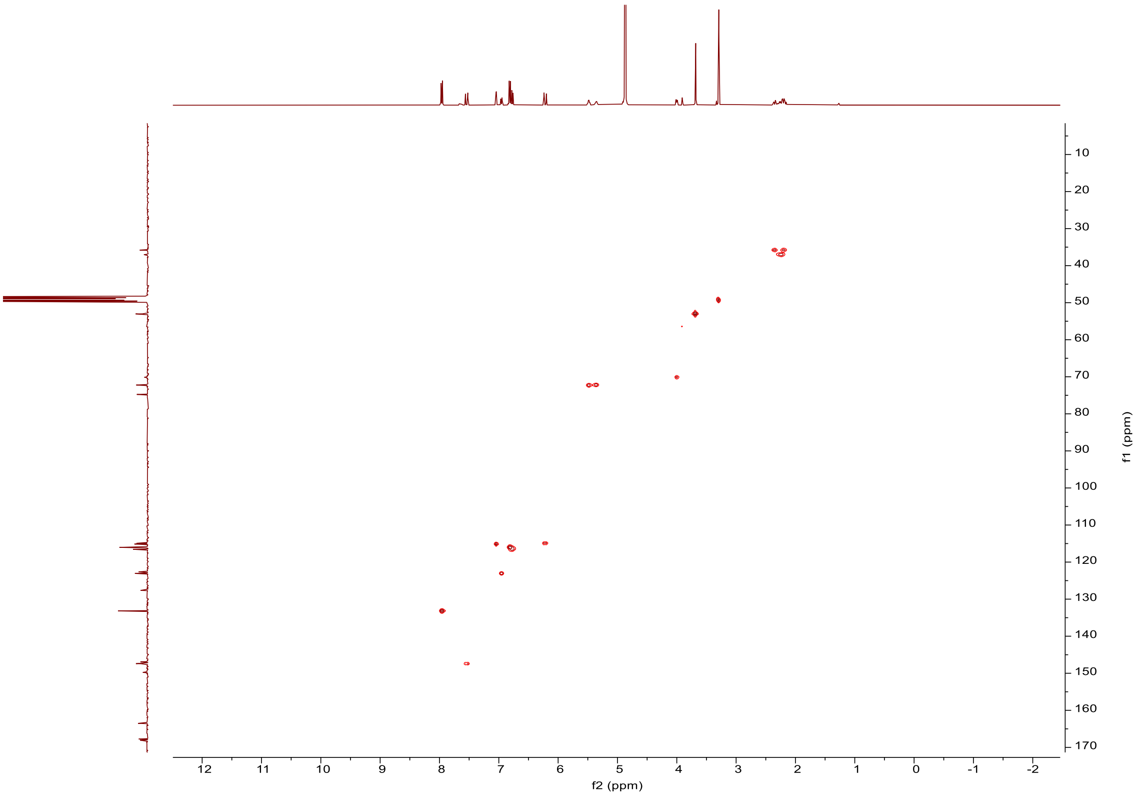

Figure S6. HMQC spectrum of **5**

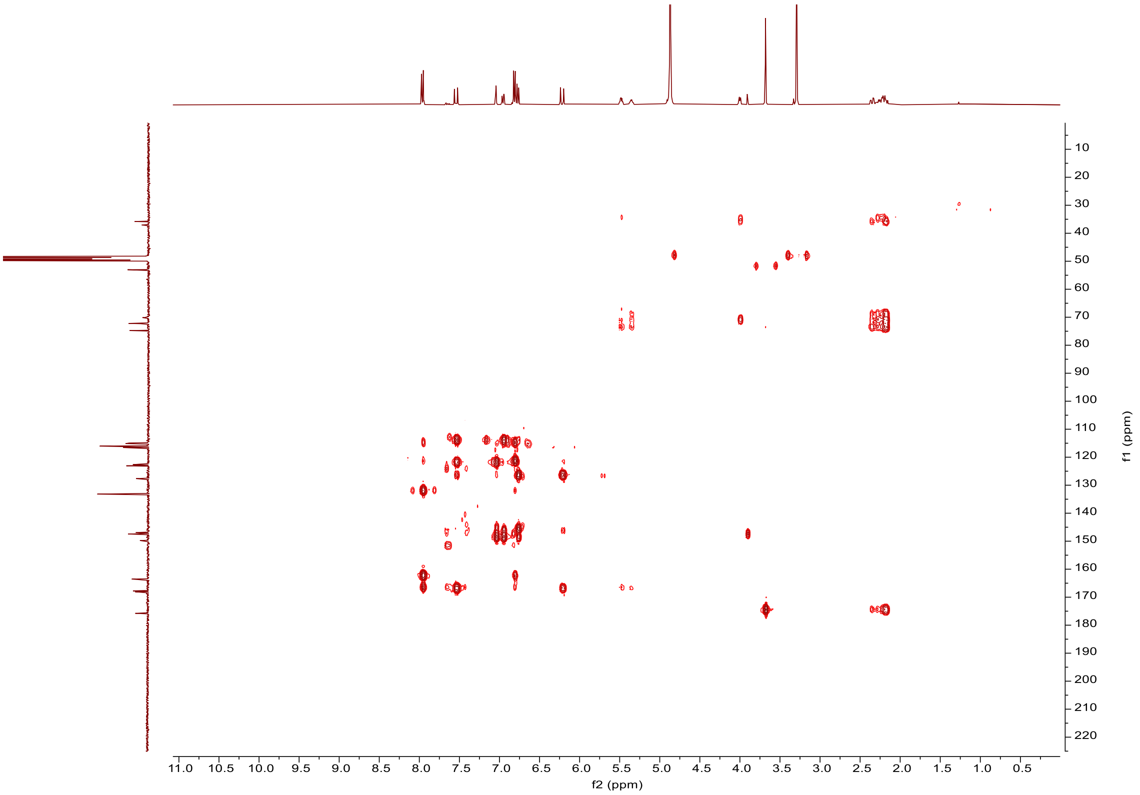

Figure S7. HMBC spectrum of **5**

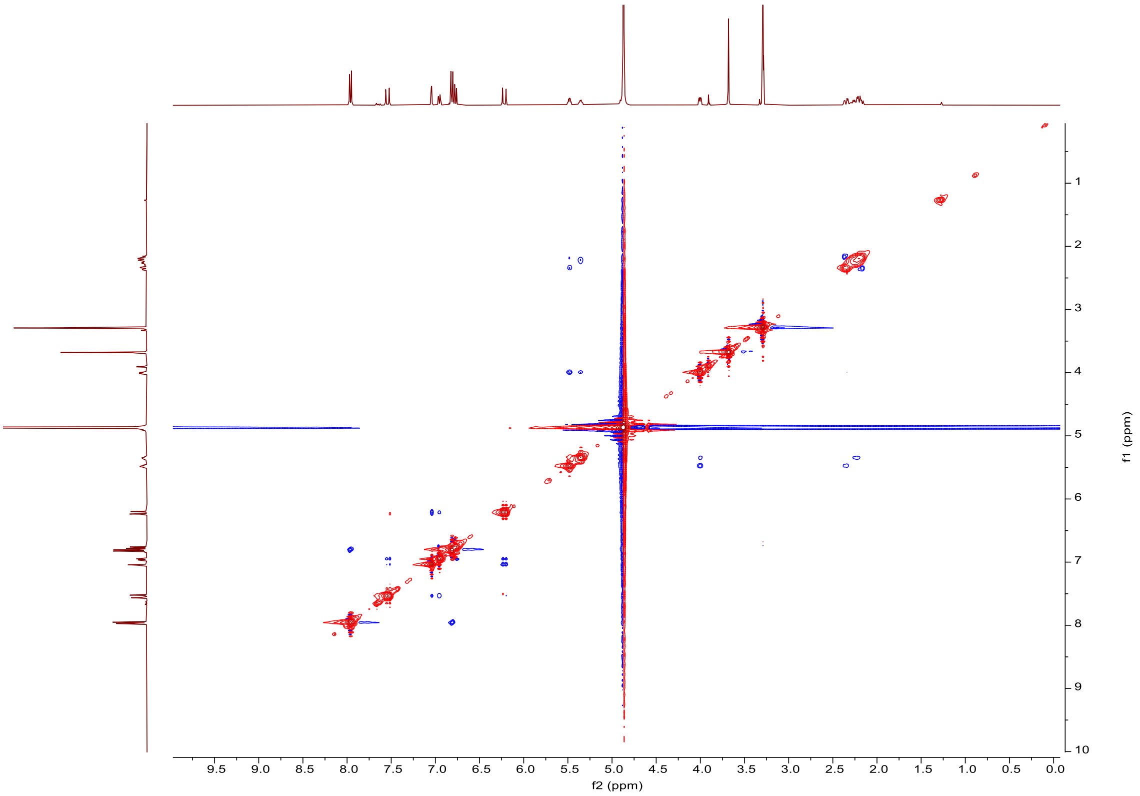

Figure S8. NOESY spectrum of **5**

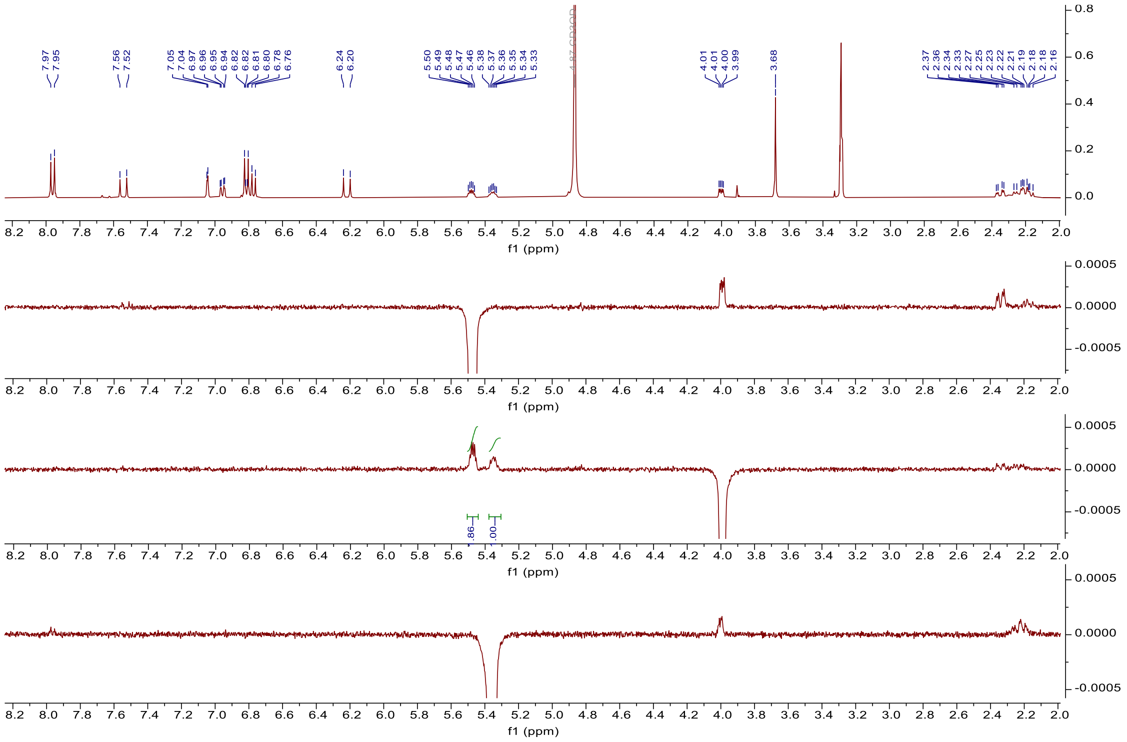

Figure S9. Selective 1D NOE spectrum of **5**

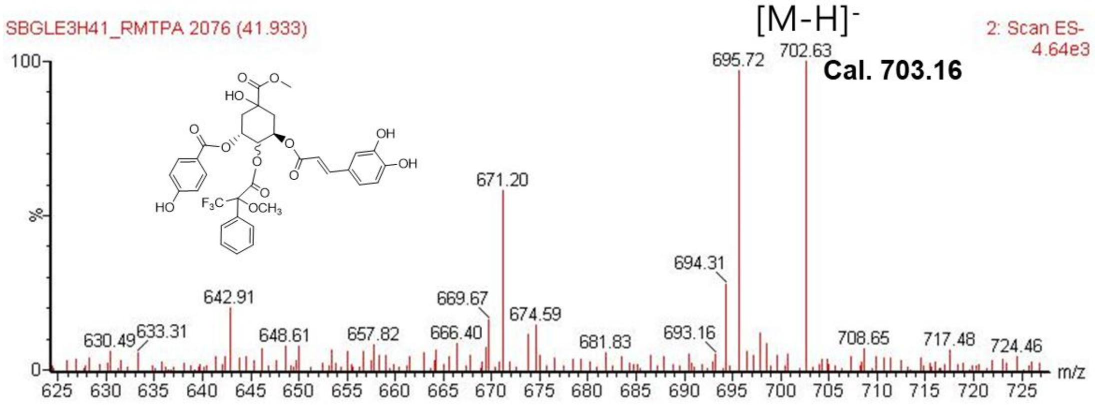

Figure S10. Low mass spectrometry of MTPA ester of compound **5**.

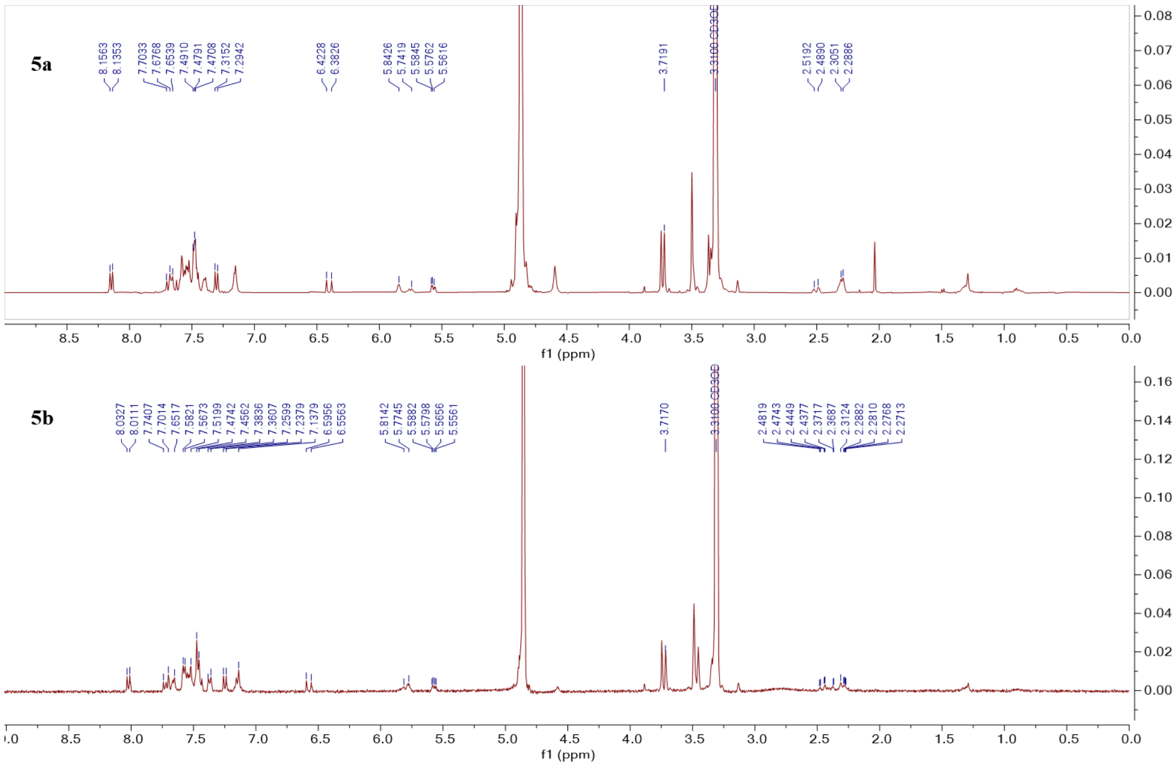

Figure S11. ^{1}H NMR spectra of *S*- and *R*-MTPA derivatives (**5a** and **5b**)

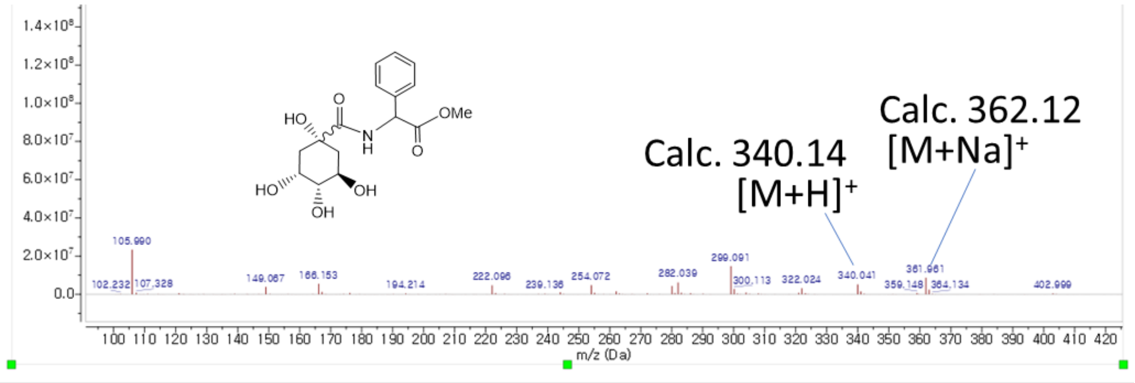

Figure S12. Low mass spectrometry of PGME ester of compound **5**

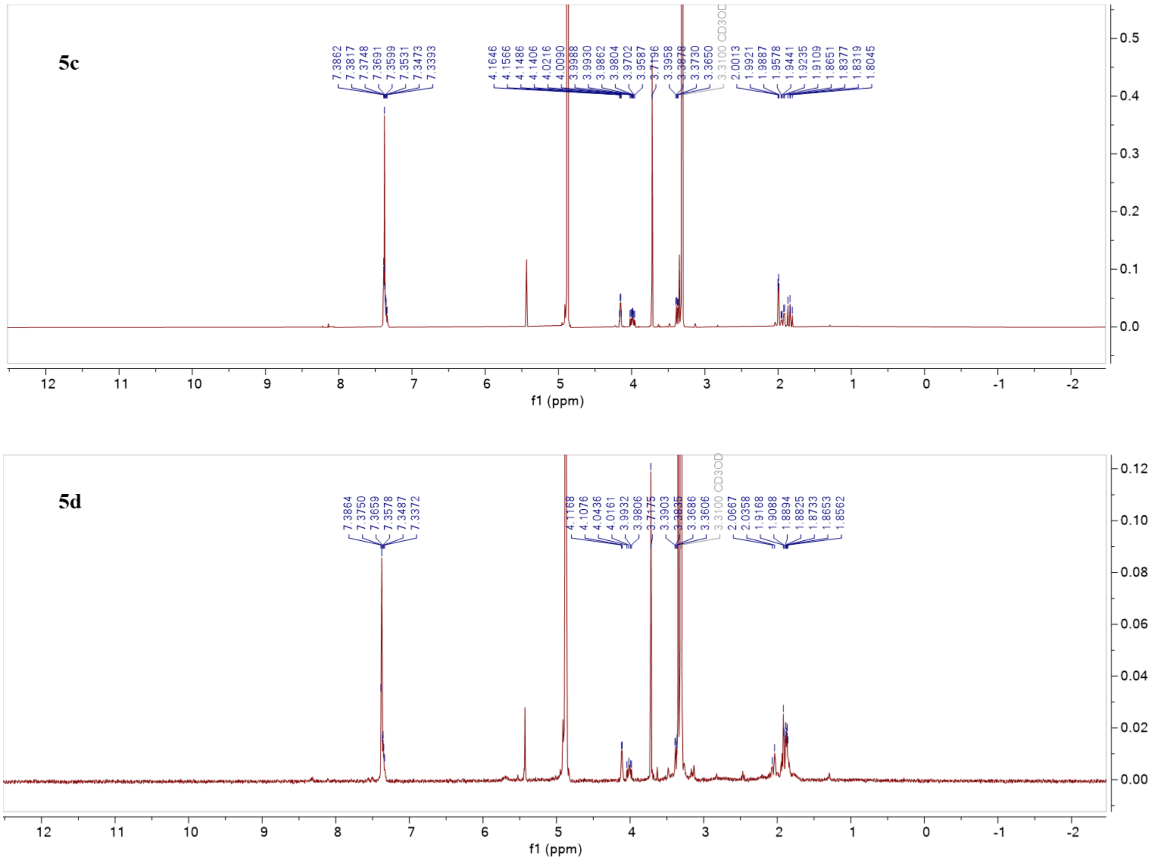

Figure S13. ^{1}H NMR spectra of *S*- and *R*-PGME amide derivatives (**5c** and **5d**).

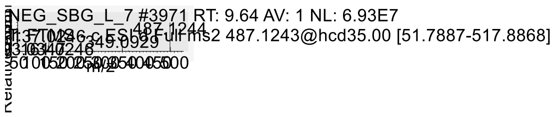

Figure S14. High mass spectrometry of **7**

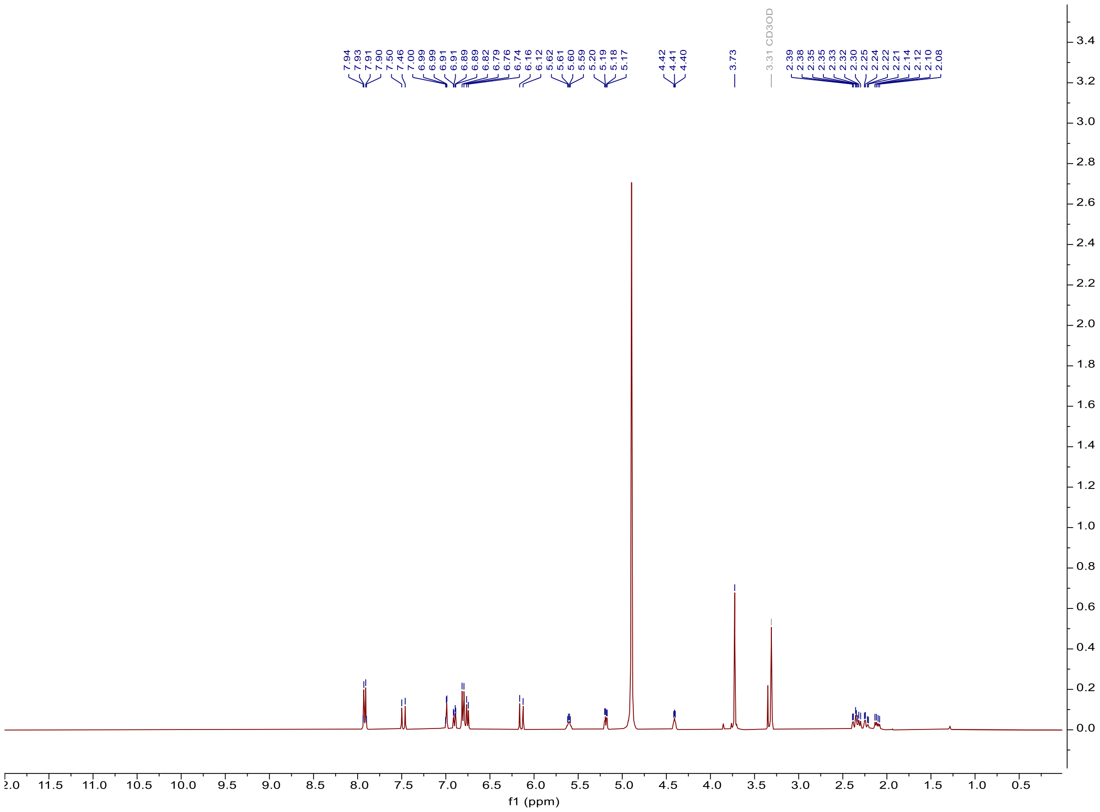

Figure S15. ^{1}H-NMR (400 MHz, CD_3OD) spectrum of **7**

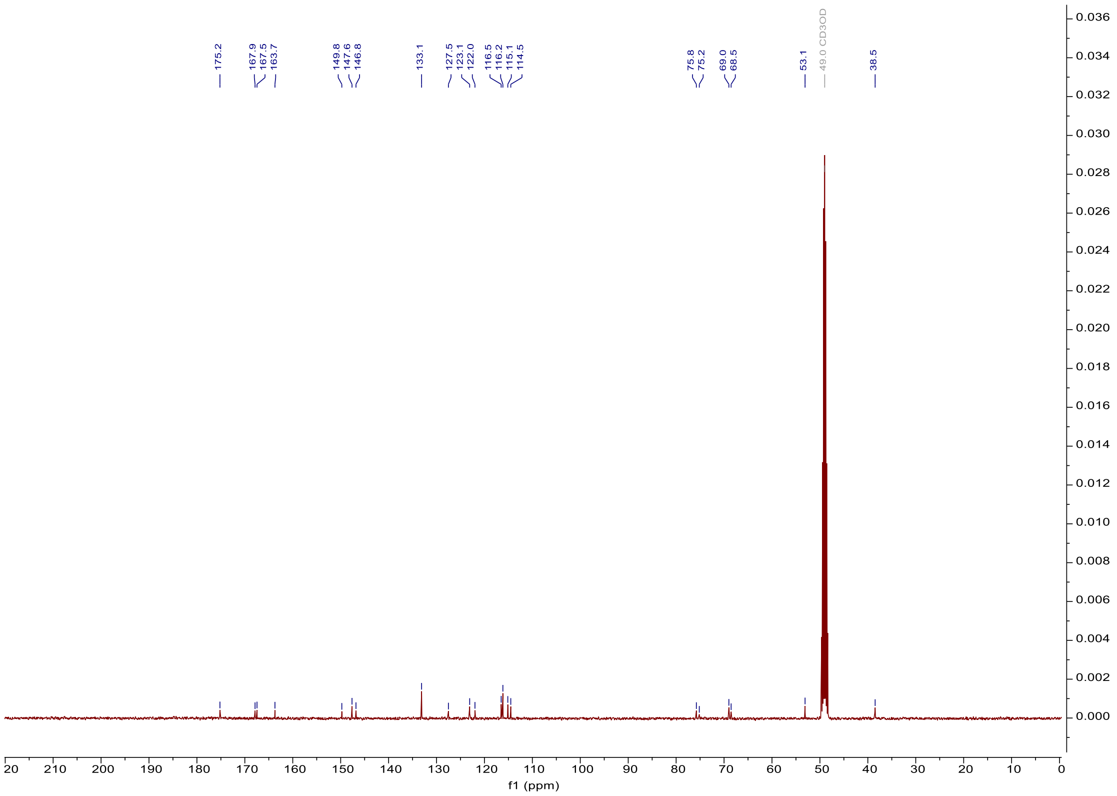

Figure S16. ^{13}C-NMR (100 MHz, CD_3OD) spectrum of **7**

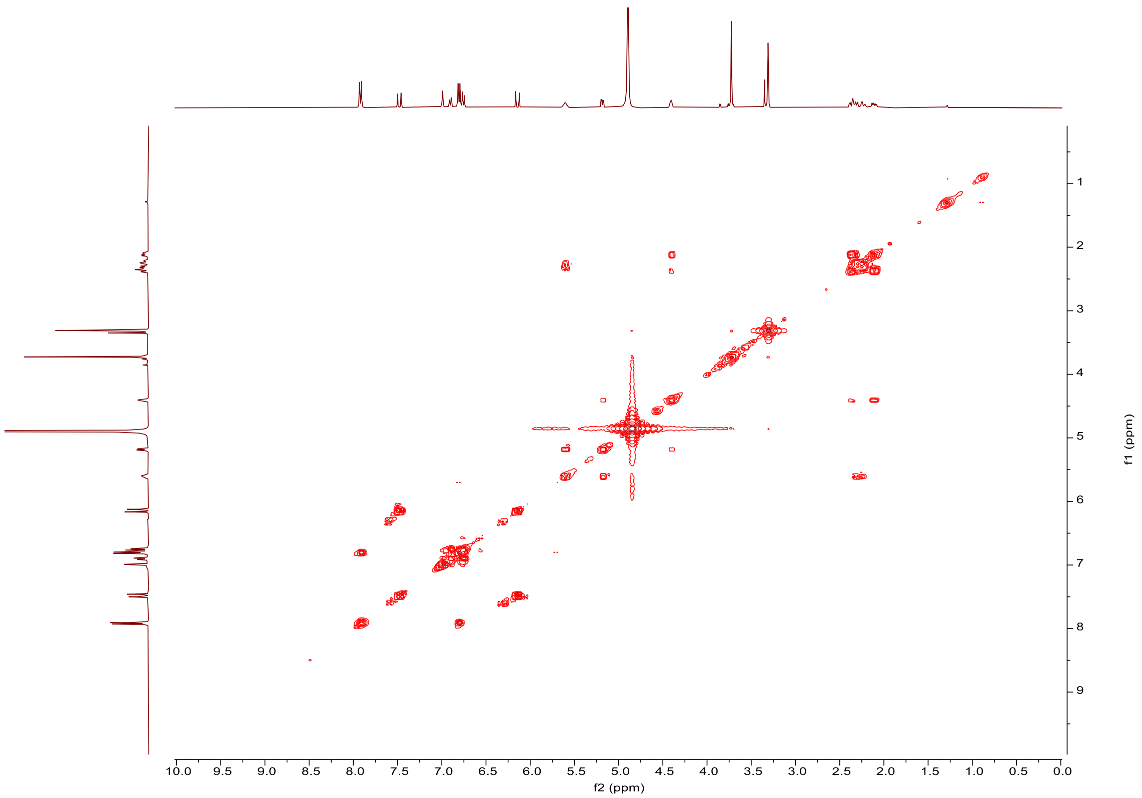

Figure S17. COSY spectrum of **7**

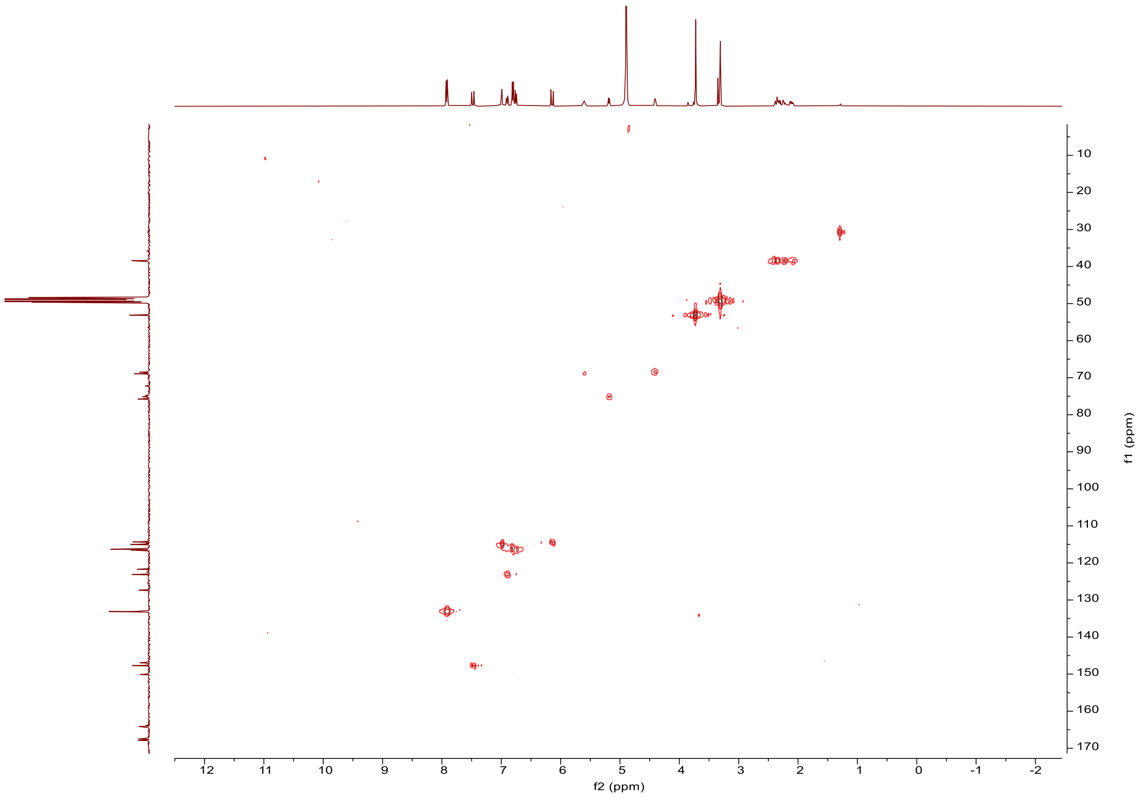

Figure S18. HMQC spectrum of **7**

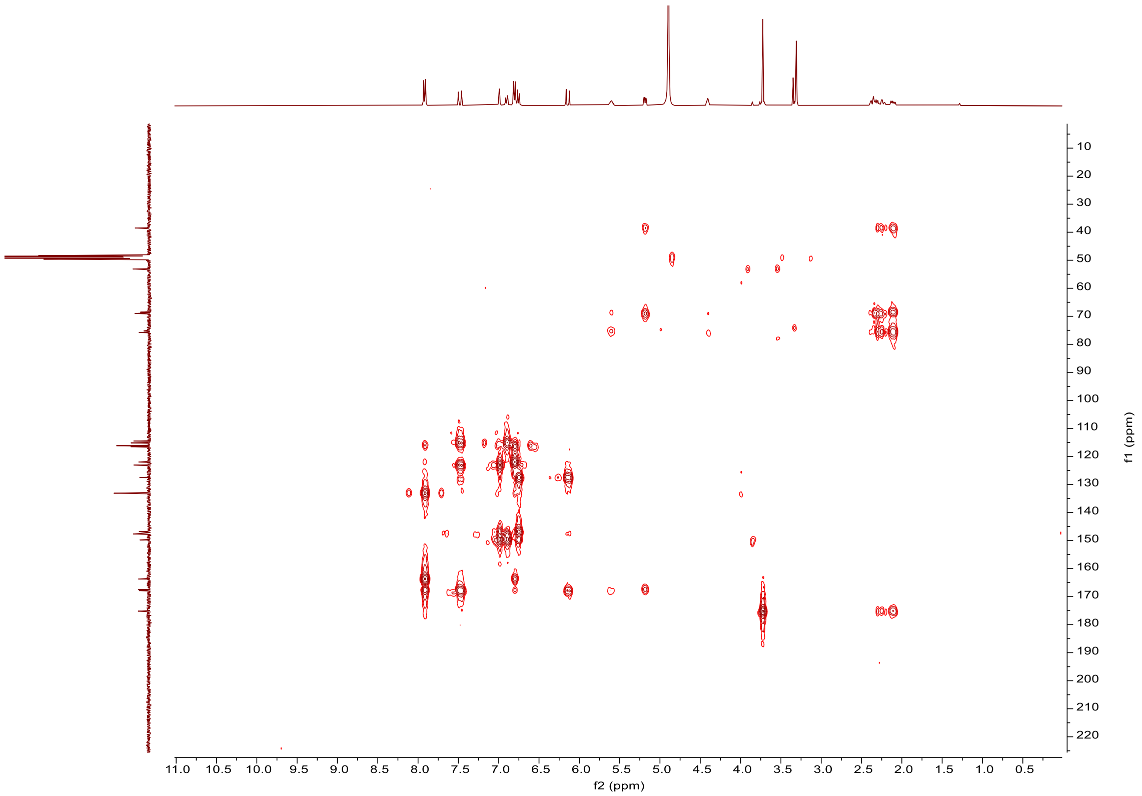

Figure S19. HMBC spectrum of **7**

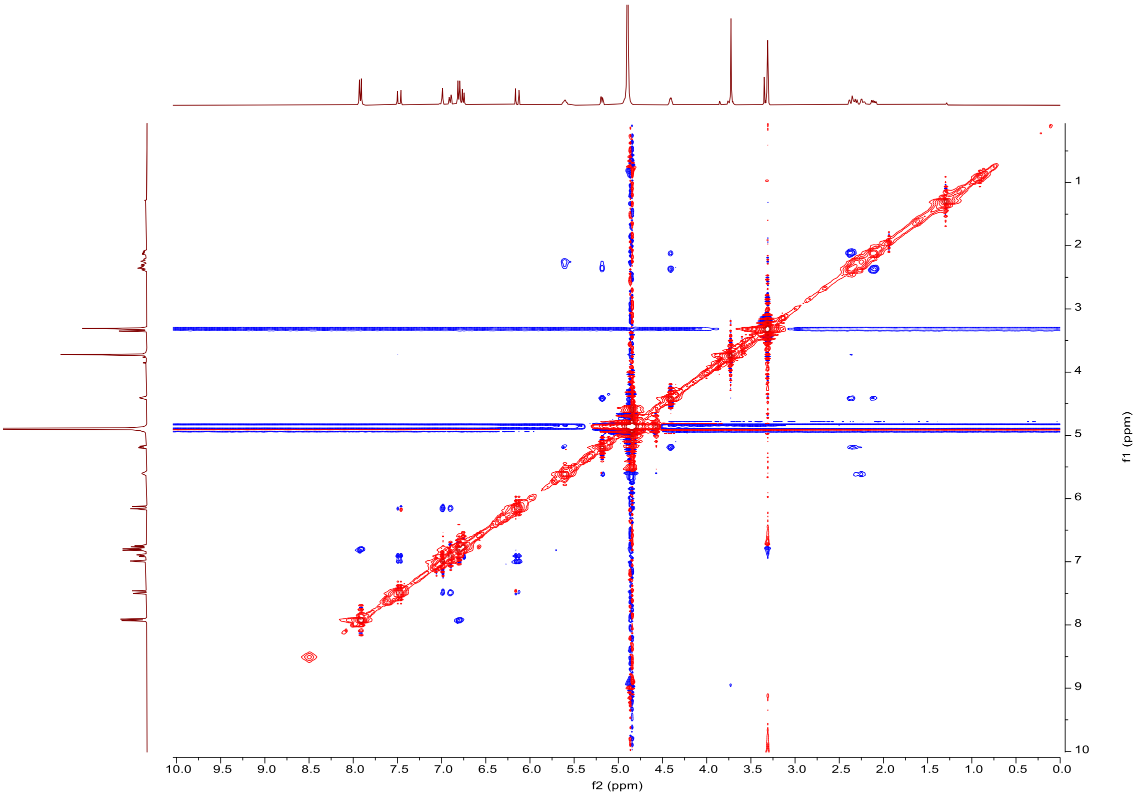

Figure S20. NOESY spectrum of **7**

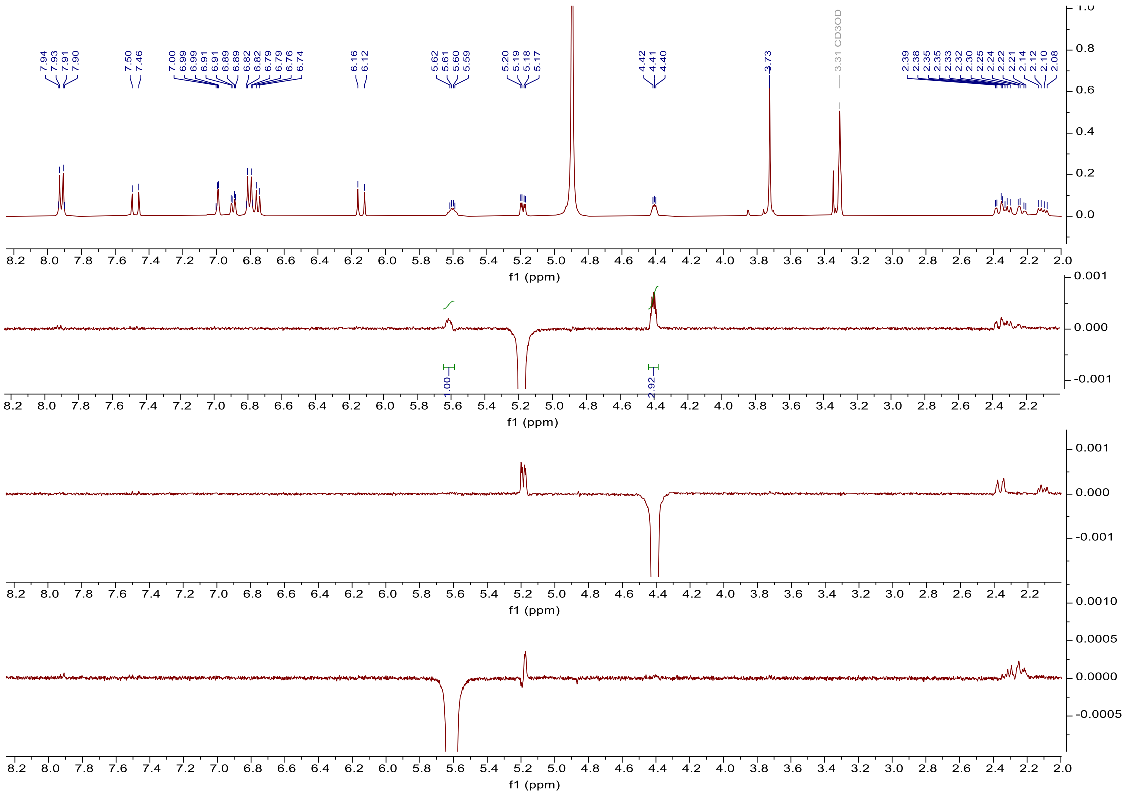

Figure S21. Selective 1D NOE spectrum of **7**

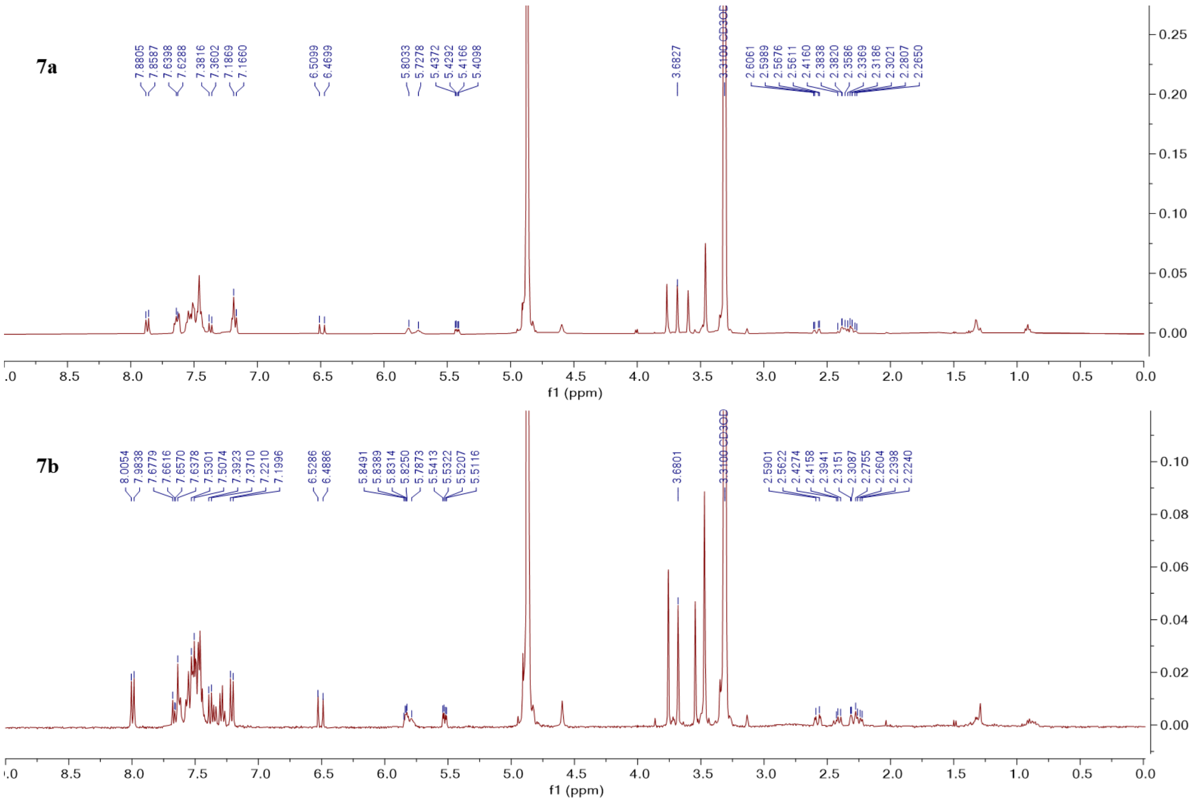

Figure S22. ^{1}H NMR spectra of *S*- and *R*-MTPA derivatives (**7a** and **7b**)

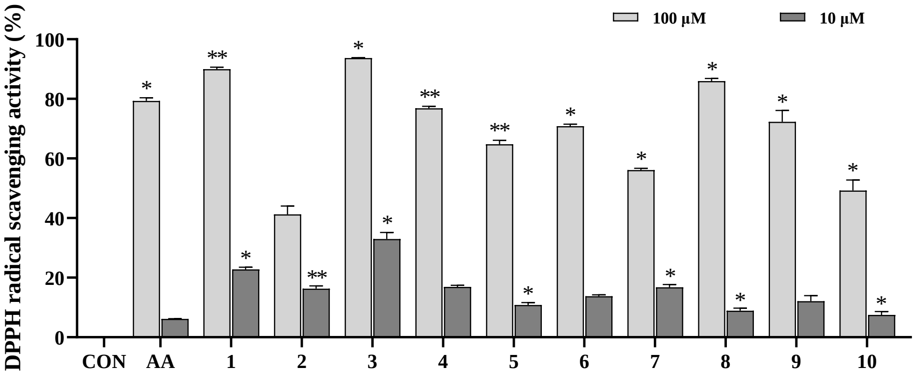

Figure S23. Antioxidant effect of compounds (**1–10**). DPPH assay was performed in triplicates. The data are represented as mean ± SD. *p < 0.05, **p < 0.01, compared to control (AA).

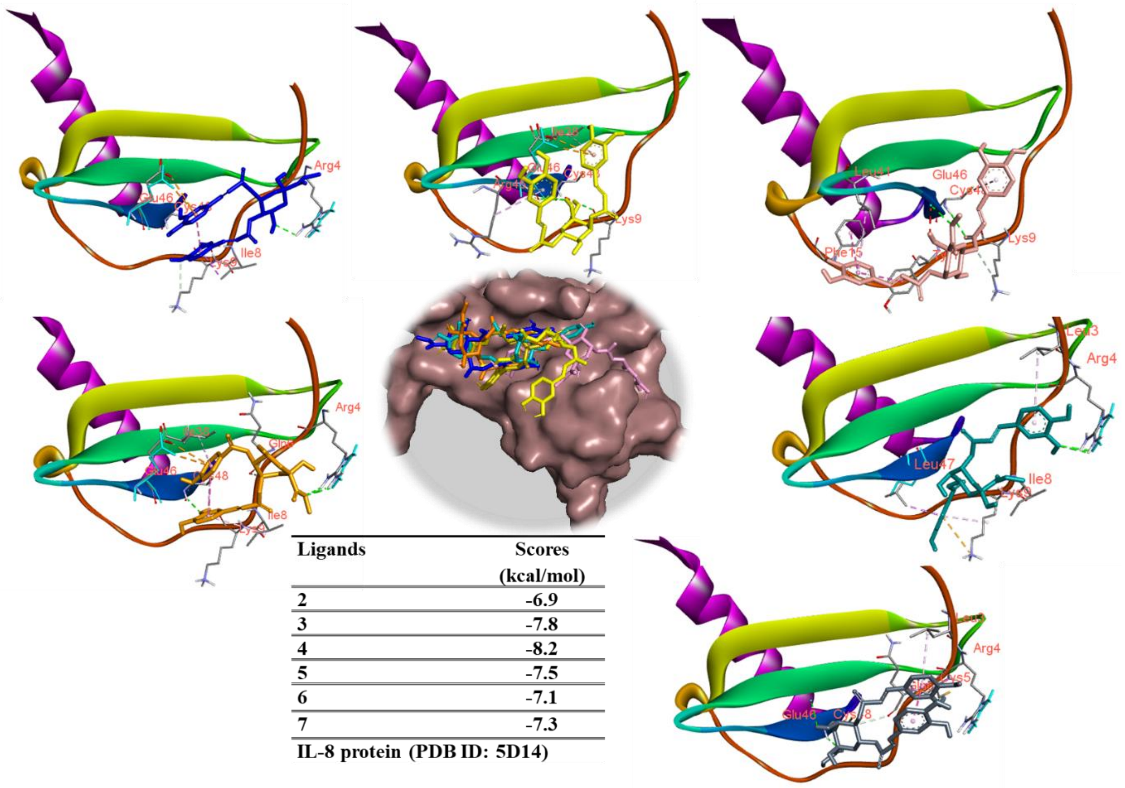

Ligands	Scores (kcal/mol)
2	-6.9
3	-7.8
4	-8.2
5	-7.5
6	-7.1
7	-7.3
IL-8 protein (PDB ID: 5D14)	

Figure S24. Interactions of **2** (blue), **3** (yellow), **4** (pink), **5** (teal), **6** (grey), **7** (orange) docked into IL-8 protein (PDB ID: 5D14) with 3D visualization.

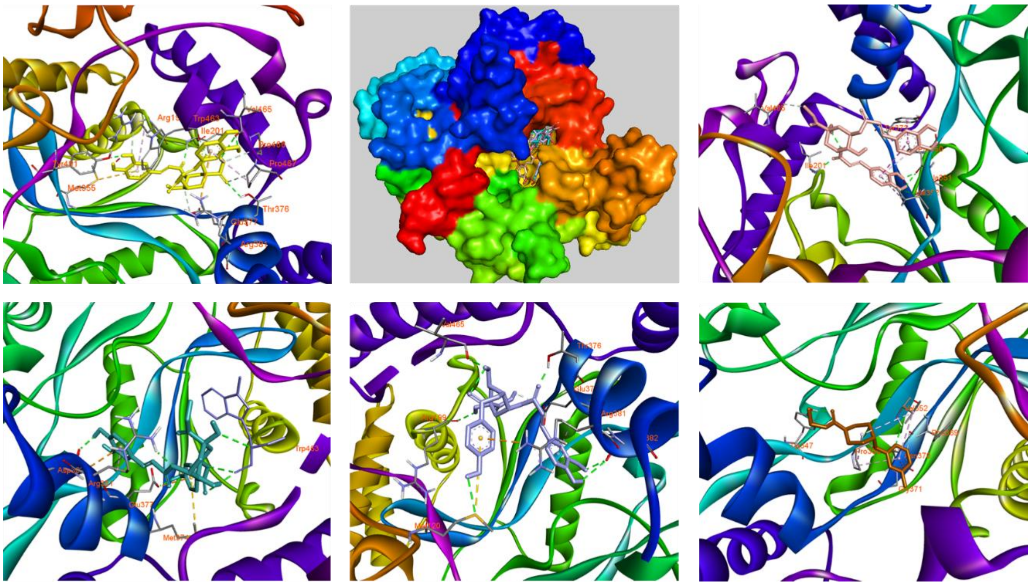

Figure S25. Interactions of **3** (yellow), **4** (pink), **5** (teal), **7** (lightblue), and AT2 (brown) docked into iNOS protein (PDB ID: 3E7G) with 3D visualization.

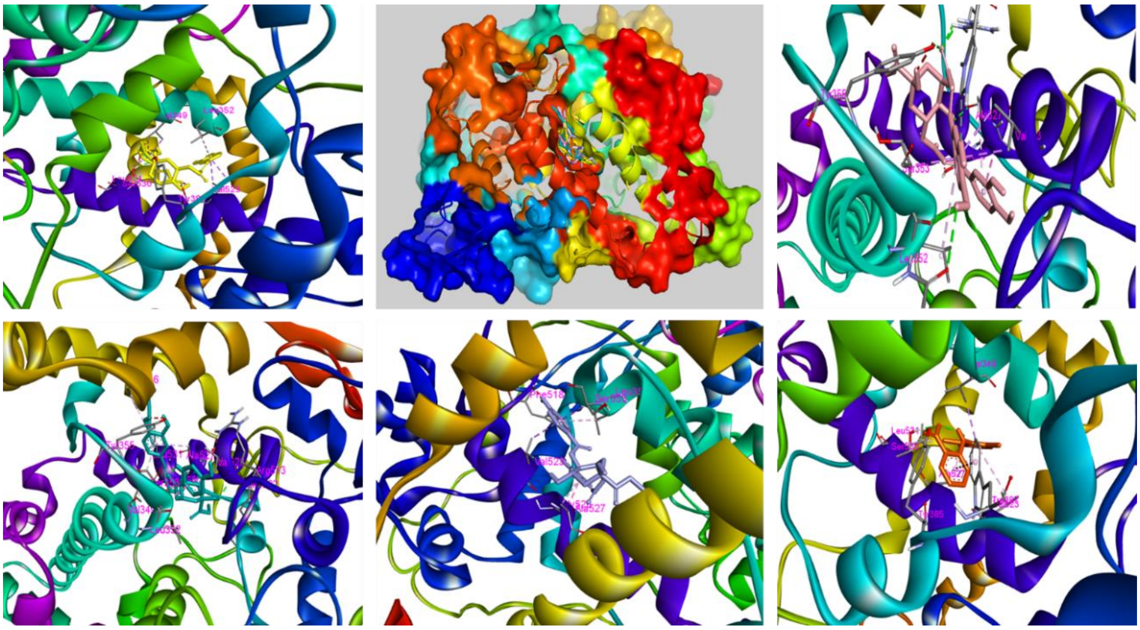

Figure S26. Interactions of **3** (yellow), **4** (pink), **5** (teal), **7** (lightblue), and JMS (orange) docked into COX-2 protein (PDB ID: 5IKQ) with 3D visualization.

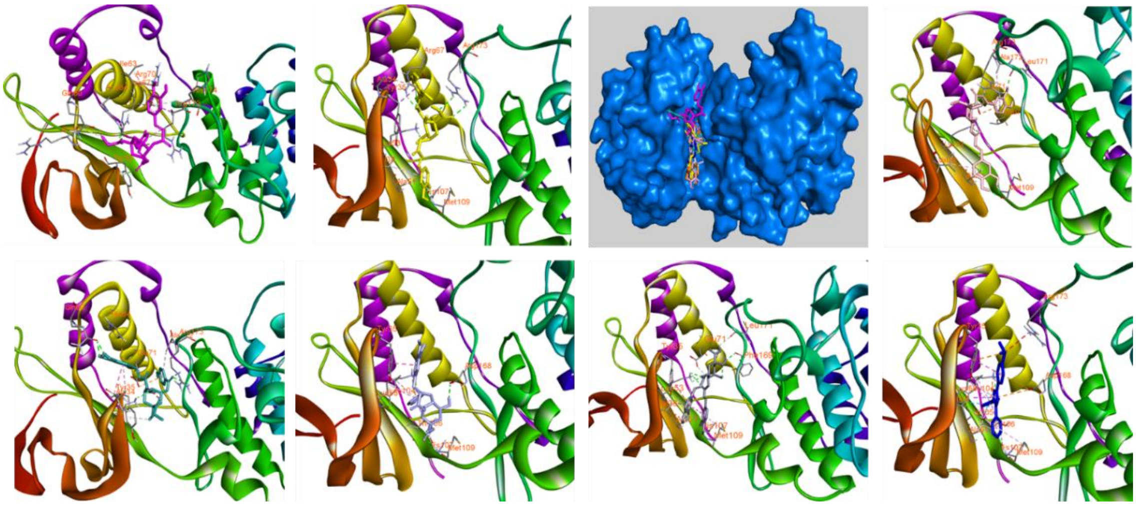

Figure S27. Interactions of **2** (magenta), **3** (yellow), **4** (pink), **5** (cyan). **7** (violet), **8** (grey), and SB203580 (blue) docked into MAP Kinase P38 protein (PDB ID: 1A9U) with 3D visualization.
